# PGC1 alpha coactivates ERG fusion to drive antioxidant target genes under metabolic stress

**DOI:** 10.1038/s42003-022-03385-x

**Published:** 2022-05-04

**Authors:** Aiindrila Dhara, Imlimaong Aier, Ankush Paladhi, Pritish Kumar Varadwaj, Sumit Kumar Hira, Nirmalya Sen

**Affiliations:** 1 Molecular oncology laboratory, S.N. Bose innovation centre, University of Kalyani, Kalyani, West Bengal 741235 India; 2 Department of Bioinformatics & Applied Sciences, Indian Institute of Information Technology—, Allahabad, Uttar Pradesh 211012 India; 3 Cellular Immunology Laboratory, Department of Zoology, The University of Burdwan, Burdwan, West Bengal 713104 India

**Keywords:** Prostate cancer, Cancer

## Abstract

The presence of *ERG* gene fusion; from developing prostatic intraepithelial neoplasia (PIN) lesions to hormone resistant high grade prostate cancer (PCa) dictates disease progression, altered androgen metabolism, proliferation and metastasis^1–3^. ERG driven transcriptional landscape may provide pro-tumorigenic cues in overcoming various strains like hypoxia, nutrient deprivation, inflammation and oxidative stress. However, insights on the androgen independent regulation and function of ERG during stress are limited. Here, we identify PGC1α as a coactivator of ERG fusion under various metabolic stress. Deacetylase SIRT1 is necessary for PGC1α-ERG interaction and function. We reveal that ERG drives the expression of antioxidant genes; *SOD1* and *TXN*, benefitting PCa growth. We observe increased expression of these antioxidant genes in patients with high ERG expression correlates with poor survival. Inhibition of PGC1α-ERG axis driven transcriptional program results in apoptosis and reduction in PCa xenografts. Here we report a function of ERG under metabolic stress which warrants further studies as a therapeutic target for ERG fusion positive PCa.

## Abstract (web summary)

PGC1α acts as a co-activator of the ERG transcription factor during metabolic stress resulting in antioxidant functionsand inhibition of the PGC1α-ERG driven transcriptional program reduces prostate cancer growth by inducing ROS mediated apoptosis.

## Introduction

E-Twenty Six transcription factor *ERG*; exists as a fusion oncogene; in many cancers including myeloid leukemia, Ewing sarcoma, and prostate cancer (PCa)^4–7^. Physiologically, temporal tissue-specific expression of *ERG* is important for hematopoiesis, angiogenesis, and valve development^8–10^. Although reported in ~50% of PCa with connection to aggressiveness and castration resistance (CrPC), the role of * TMPRSS2-ERG* fusion gene in driving CrPC is not addressed thoroughly^1–3,7,11^. Recent studies of androgen receptor (AR) independent PCa with altered transcriptional circuits strengthen the possibility of ERG fusion-driven programs^12–14^. Like many cancers, metabolic programs associated with altered transcription is an emerging hallmark for PCa and accentuate the requirement of specific transcription factors and nutrients during disease progression^15–17^. In this context, metabolic profiling of ERG fusion-positive PCa patients exhibits altered metabolism of glucose, citrate, and fatty acid associated with high Gleason score^15,18–20^. However, the signals that drive ERG in absence of androgen or under nutrient stress are not well known. Recently, *PPARGC1A* (PPAR gamma Coactivator 1 alpha/PGC1α) a well-known coactivator has been implicated in PCa oncogenesis via coactivation of important transcription factors like *AR, ERR-*α^21–23^. PGC1α is a metabolically inducible coactivator that can transactivate various transcription factors in a tissue-dependent manner under metabolic stress^24^ Interestingly, PGC1α can coactivate a plethora of transcription factors in cancers including PCa, thereby regulating cancer progression^25–27^. Nonetheless, the behavior of PGC1α under nutrient deprivation has not been studied in the context of PCa progression. We speculate that metabolically induced PGC1α may transactivate ERG fusion-driven programs under metabolic stress.

In this study, we found that PGC1α can interact and coactivate ERG under various metabolic stress in the presence of SIRT1 deacetylase and drives an antioxidant function. We found antioxidant target genes of ERG fusion; namely *SOD1* and *TXN* resulting in reactive oxygen species (ROS) clearance and survival. Abrogation of *PGC1*α*, ERG*, or antioxidant genes *SOD1* and *TXN* results in ROS-mediated apoptosis during metabolic stress. PCa xenografts show tumor regression and apoptosis in absence of PGC1α. Together our study identifies ERG- PGC1α axis during metabolic stress which might be crucial for PCa progression.

## Results

### PGC1α acts as a coactivator for ERG fusion under metabolic stress

**Fig. 1:**
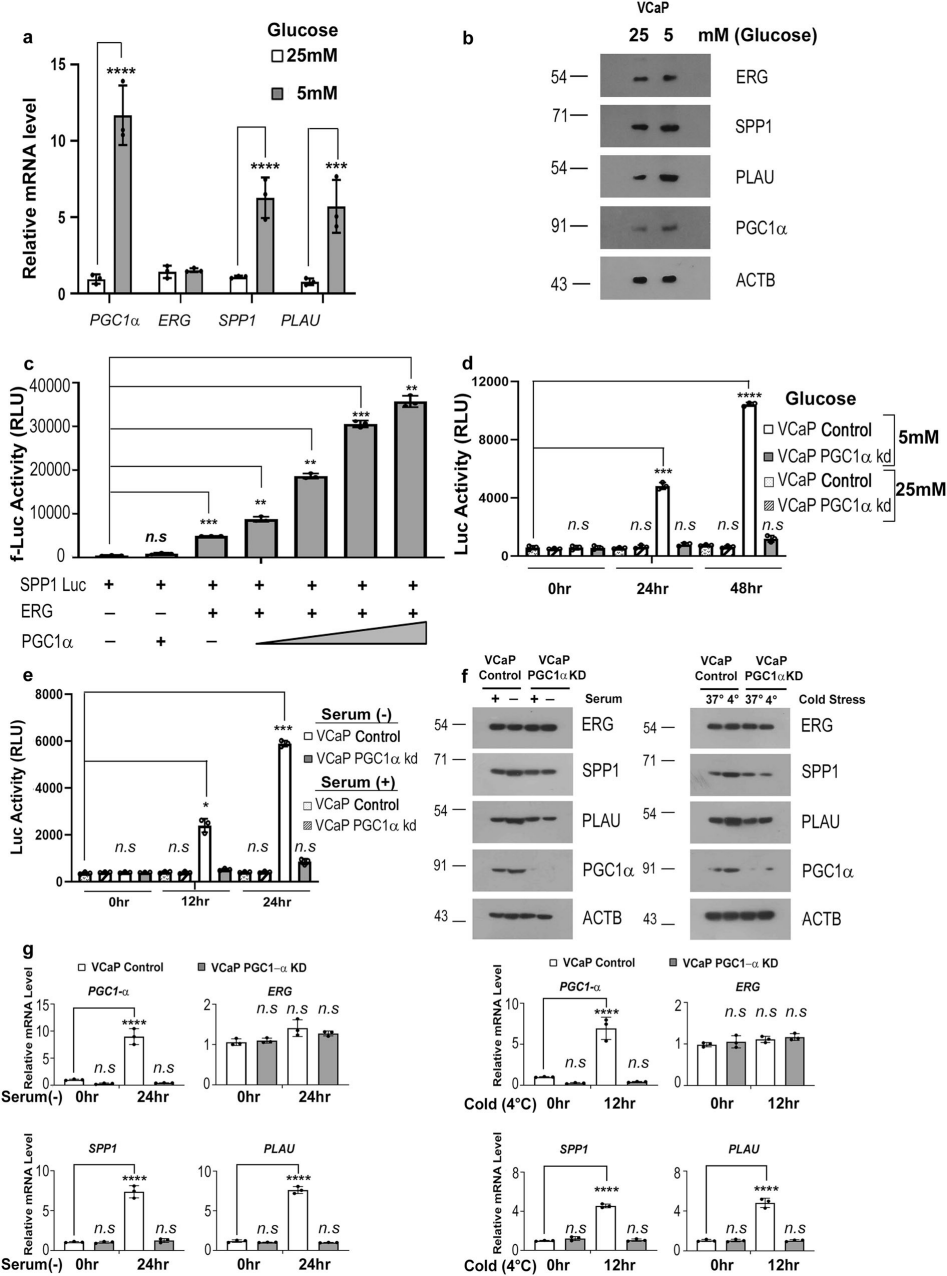
PGC1α acts as a coactivator for ERG fusion under metabolic stress. **a** VCap Cells were treated for 24 h. Relative transcript levels of genes were analyzed using qRT-PCR. **b** VCaP cells were treated for 24 h and the whole-cell extract was immunoblotted using indicated antibodies. **c** PC3 cells were cotransfected as indicated. Luciferase assay was performed post 24 h of transfection and relative firefly luciferase activity was plotted. **d** VCaP control (Scrambled shRNA) or VCAP PGC1α KD (PGC1α shRNA#1) stable cells transfected with SPP1 luciferase were glucose deprived(5 mM) or cultured under basal condition(25 mM) post-transfection as indicated. Luciferase assay was performed and relative firefly luciferase activity was plotted. **e** VCaP control or VCAP PGC1α KD stable cells transfected with SPP1 luciferase were serum-deprived or cultured under basal condition post-transfection as indicated. Luciferase assay was performed and relative firefly luciferase activity was plotted. **f** VCaP control or VCAP PGC1α KD stable cells were serum-starved (Left panel) or received cold stress (4 °C, Right panel) as indicated for 24 h and the whole-cell extract was immunoblotted using indicated antibodies. **g** VCaP control or VCAP PGC1α KD stable cells were serum-starved (Left panel) or received cold stress (4 °C, Right panel) as indicated for 24 h. Relative transcript levels of genes were analyzed using qRT-PCR. Experiments were performed as biological triplicates (mean ± SD). Two-way ANOVA with multiple comparison test used for statistical significance of 1a, 1g. For 1c, d, e; one-way ANOVA with Dunnett’s multiple comparison test used for statistical significance where ****p < .0001, ***p = 0.0001, **p = .001, *p = .01, n.s not significant.

PGC1α is known to get induced and activated under various metabolic stress and hence act as a coactivator^24,27–29^. We tested if PGC1α affects ERG function under metabolic stress; like glucose or serum deprivation. We deprived VCaP cells harboring ERG fusion of glucose (5 mM) as described previously^30^ and checked for expression of *ERG, PGC1*α and verified ERG targets *SPP1*^31^ (secreted phosphoprotein 1/Osteopontin) and *PLAU*^3^ (plasminogen activator, urokinase). We found increased expression of *PGC1*α without any alteration of *ERG*. Interestingly, we observed an increased expression of ERG target genes, *SPP1* and *PLAU* under glucose stress (Fig. 1a, b). We have used, AICAR (*N*^1^-(β-d-Ribofuranosyl)-5-aminoimidazole-4-carboxamide), to mimic glucose deprivation in VCaP cells. Post-treatment with AICAR showed similar alterations in the expression of *ERG* and its target genes (Fig. S1a, b). To test the transactivation function of PGC1α over ERG during metabolic stress, we used luciferase reporter tagged SPP1 promoter (SPP1-Luc), in PC3 cells that lack ERG fusion^31^. Introducing exogenous *ERG* along with an increasing amount of *PGC1*α in PC3 resulted in increased SPP1-Luc activity (Fig. 1c). We speculate that overexpression of PGC1α possibly mimics induced PGC1α levels found during metabolic stress resulting in SPP1 luciferase activity. However, in absence of ERG, induced or exogenous levels of PGC1α couldn’t rescue SPP1 luciferase activity indicating PGC1α works by transactivating ERG (Fig. S1c). We verified three different PGC1α shRNA and used VCaP cells with stably silenced PGC1α expression containing shRNA#1^30^ to check the effects of PGC1α over ERG fusion under metabolic stress (Fig. S1d). Glucose deprivation was able to increase SPP1-Luc activity in VCaP control cells but not in VCaP PGC1αKD cells, indicating PGC1α is important for the transactivation of endogenous ERG (Fig. 1d). Basal glucose concentration (25 mM) was unable to induce PGC1α and hence fail to activate ERG-mediated SPP1 luciferase activity (Fig. 1d). We observed a similar PGC1 mediated transactivation function only under glucose deprivation in another ERG containing cell line Colo320 but not in PGC1αKD Colo320 cells or in basal conditions (Fig. S1e). Additionally, AICAR treatment showed coactivation of SPP1-Luc promoter in VCaP control cells in contrast to PGC1αKD cells (Fig. S1f). We further checked the expression of ERG target genes in control and PGC1αKD; VCaP and Colo320 cells. As reported previously^24^, PGC1α expression is induced under glucose deprivation resulting in activation of ERG target genes SPP1 and PLAU. Glucose deprivation in VCaP and Colo320 PGC1αKD cells did not show any induction of ERG target genes (Fig. S1g). AR-mediated signaling is an important regulator of ERG fusion mediated transcriptional function. To understand the effects of AR modulation during metabolic stress, we treated glucose-deprived VCaP cells with AR antagonist; Flutamide and measured SPP1-Luc activity. As compared to control cells, we observe minimal decrement in SPP1 activity in the presence of Flutamide; indicating PGC1α can trigger ERG transactivation independent of androgen response (Fig. S1h). We also checked if the PGC1α coactivation function over ERG is affected by the androgen analog; R1881. Interestingly, we found that R1881 addition to VCaP control cells further enhanced SPP1-Luc activity suggesting a possible synergistic effect as reported by others (Fig. S1i)^21^. However, R1881 stimuli in VCaP PGC1αKD cells couldn’t activate SPP1-Luc at 24 h, indicating PGC1α is important for the coactivation of ERG during metabolic stress (Fig. S1i). As reported by others^32^, we did not observe any upregulation of *PGC1*α mRNA levels in VCap cells upon treatment with R1881 at normal glucose concentration (Fig. S1j). We checked the effects of other metabolic stress like serum deprivation and cold stress in the coactivation of ERG. Indeed, serum deprivation and cold stress-induced PGC1 coactivation function subsequently resulted in ERG-mediated activation of the SPP1-Luc promoter (Figs. 1e and S1k). The basal condition of 10% serum couldn’t induce PGC1 and subsequently failed to show any SPP1 luciferase activity (Fig. 1e). We checked for ERG target gene expression in VCaP cells (Control or PGC1αKD) under serum deprivation or cold stress and observed induction in control cells but not in PGC1αKD cells (Fig. 1f, g). We further tested if *PGC1-β*, a homologous PGC1 family member; has overlapping functions towards transactivation of ERG. Co-transfection with *PGC1-β* did not alter the transactivation of SPP1-Luc, indicating *PGC1-β* to be non-essential for ERG transactivation functions (Fig. S1l). Together, the results indicate that only under metabolic stress PGC1α is induced and may act as a coactivator for ERG transcription factor.

### PGC1α interacts and transactivates ERG fusion

**Fig. 2:**
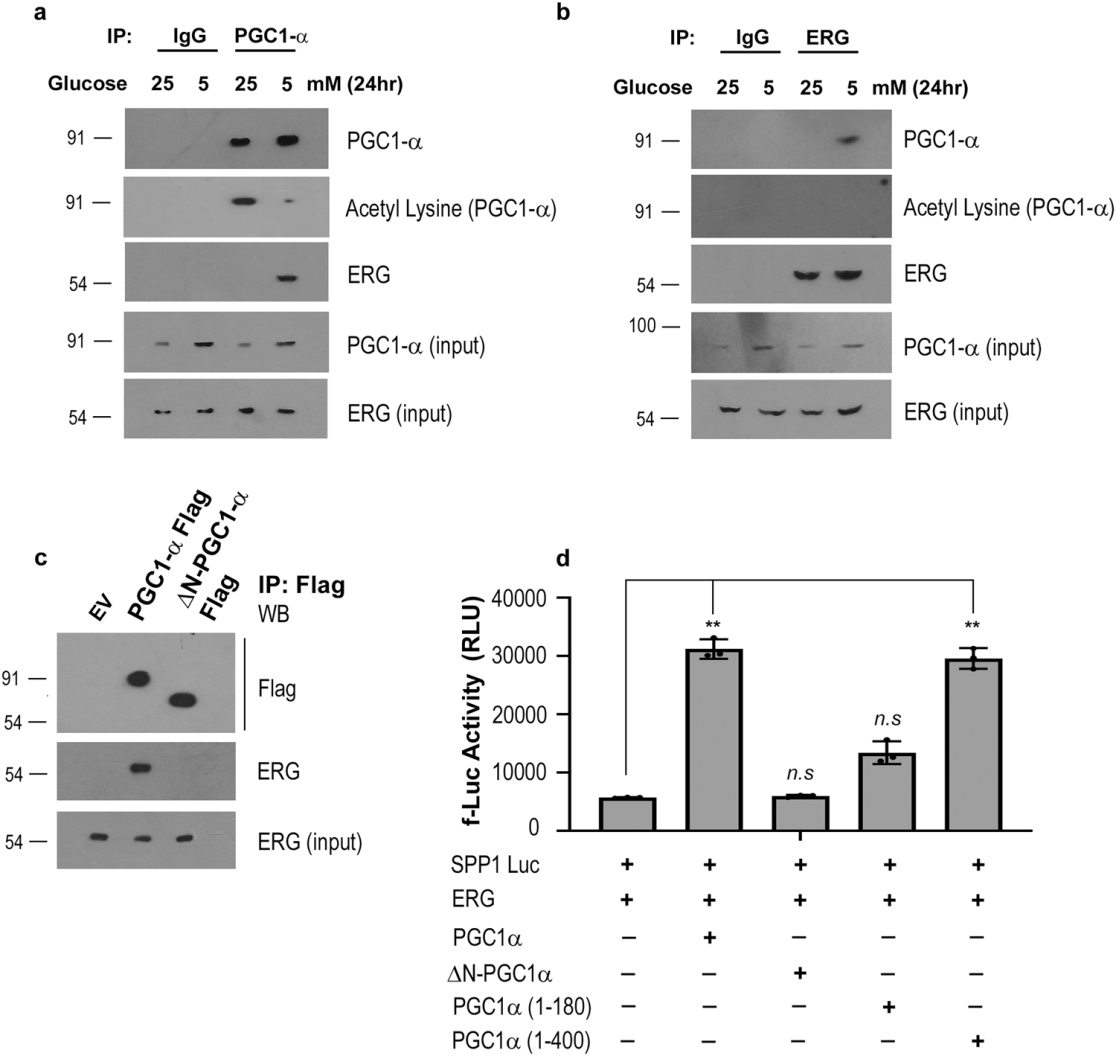
PGC1α interacts and transactivates ERG fusion. **a** VCaP cells were treated as indicated. Cells were harvested after 24 h and subjected to immunoprecipitations using anti-PGC1α antibody or anti-IgG antibody, and western blots were performed for the indicated proteins. **b** VCaP cells were treated as indicated. Cells were harvested after 24 h and subjected to immunoprecipitations using anti-ERG antibody or anti-IgG antibody, and western blots were performed for the indicated proteins. **c** VCaP cells were transfected with either vector control(EV), PGC1α Flag, or ΔN-PGC1α Flag as indicated. Cells were harvested 24 h post-transfection and subjected to immunoprecipitations using anti-FLAG antibody and western blots were performed for the indicated proteins. **d** PC3 cells were cotransfected as indicated. Luciferase assay was performed 24 h post-transfection and relative firefly luciferase activity was plotted. Experiments were performed as biological triplicates (mean ± SD). One-way ANOVA with Dunnett’s multiple comparison test was used for statistical significance of 2d where **p = 0.001.

In order to act as a coactivator, PGC1α needs to interact with the ERG transcription factor. PGC1 family of proteins are known to interact with various transcription factors under nonbasal conditions, resulting in transactivation of such factors^25–27^. Under metabolic stress, PGC1α is activated by deacetylation and is induced at mRNA and protein levels for interaction^27–29,33^. For endogenous interaction, VCaP cells were glucose deprived for 24 h and immunoprecipitation of PGC1α protein was performed. We found that induced PGC1α interacted with endogenous ERG during glucose starvation but not under basal conditions (Fig. 2a). A reverse IP on glucose starved VCaP cells with ERG antibody showed that endogenous ERG interacts with PGC1α during metabolic stress (Fig. 2b). Interestingly, we observed that ERG interacts with a deacetylated form of PGC1α but not with the acetylated PGC1α, suggesting that an induced and activate form of PGC1 formed during metabolic stress is capable of interaction (Fig. 2a, b). We confirmed the interaction using overexpression studies in VCaP and PC3 cells and found that ERG interacts with a deacetylated form of exogenously expressed PGC1α (Fig. S2a, b). We found similar endogenous interaction during serum deprivation in VCaP cells between ERG and PGC1α (Fig. S2c). To understand if the PGC1α transactivation domain (1–180 amino acid)^34^ is necessary for interaction with ERG protein, we performed interaction studies using ectopic ΔN-PGC1α FLAG (PGC1 lacking 1–180 amino acid) in VCaP cells. We did not observe any pull-down of ERG protein with ΔN-PGC1α FLAG (Fig. 2c). Moreover, ΔN-PGC1α FLAG could not transactivate SPP1-Luc in PC3 cells (Fig. 2d). We tried to rescue the transactivation function using the PGC1α 1–180 mutant but did not observe a significant restoration of the PGC1α function. To our surprise, a 1–400 amino acid long PGC1α mutant containing both transactivation domain and deacetylase binding domain was able to completely restore transactivation function (Fig. 2d). We found that PGC1α (1–400) was able to interact with ERG protein (Fig. S2d) and rescue SPP1 luciferase activity under glucose stress in absence of full-length PGC1α (Fig. S2e). As reported earlier^34,35^, our findings indicate that both PGC1α (1–180) and PGC1α (200–400) domains are necessary for interaction and transactivation function. We speculate that 1–400 is also an activated form of PGC1α as the full-length PGC1’s inhibitory constrains are lacking^34,35^. Further PGC1α (1–400) domain was able to induce ERG target genes in PGC1α KD cells during glucose starvation (Fig. S2f). Together, the results indicate PGC1α (1–400) domain that includes the N-terminal transactivation domain and deacetylase binding domain is necessary for interaction with ERG fusion protein and results in the transactivation of ERG during metabolic stress.

### SIRT1 mediated deacetylation of PGC1α is necessary for ERG interaction and function

**Fig. 3:**
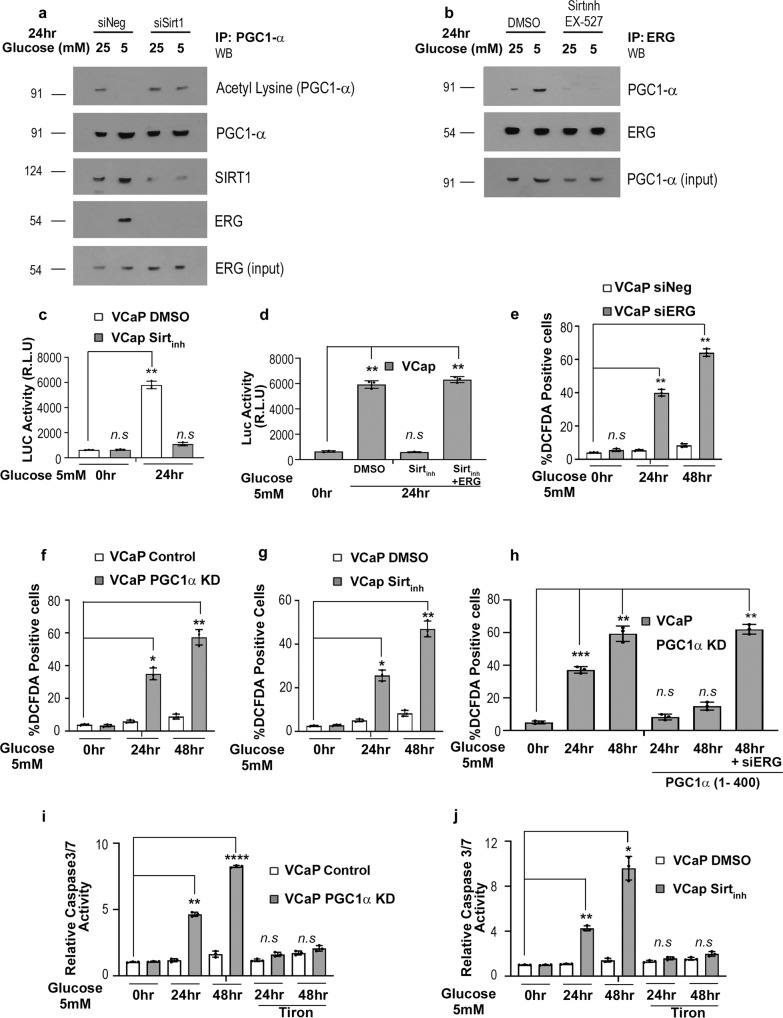
Physiological role of ERG-PGC1α axis during metabolic stress. **a** VCaP cells were transfected with either SiRNA Negative control (SiNeg) or SiRNA Sirt1. Post 24 h of transfection, cells were glucose deprived as indicated, harvested, and subjected to immunoprecipitations using anti- PGC1α antibody. Western blots were performed for the indicated proteins. **b** VCaP cells were glucose deprived and subjected to either 0.1% DMSO or 10 μM of Sirt1 inhibitor (EX-527) treatment for 24 h. Cells were harvested and subjected to immunoprecipitations using an anti-ERG antibody, and western blots were performed for the indicated proteins. **c** SPP1 luciferase transfected VCaP cells were glucose deprived and subjected to either 0.1% DMSO or 10 μM of SIRT1 inhibitor as indicated. Luciferase assay was performed and relative firefly luciferase activity was plotted. **d** SPP1 luciferase transfected VCaP cells were glucose deprived post-ERG transfection and subjected to either 0.1%DMSO or 10 μM of SIRT1 inhibitor as indicated. Luciferase assay was performed and relative firefly luciferase activity was plotted. **e** ROS levels in treated cells were measured by FACS analysis after staining with fluorescent dye DCFDA. **f** ROS levels of treated cells were measured by flow cytometry after staining with the fluorescent dye DCFDA. **g** ROS levels in glucose deprived VCaP cells treated with either 0.1% DMSO or 10 μM of Sirt1 inhibitor were measured by flow cytometry after staining with the fluorescent dye DCFDA. **h** ROS levels in treated cells post-transfection with indicated constructs were measured by FACS analysis after staining with fluorescent dye DCFDA. **i** Relative caspase3/7 activity of glucose-deprived VCaP control or VCAP PGC1α KD stable cells were measured at the indicated time. ROS scavenger; Tiron (5 mM) was added for the last 12 h as indicated. **j** Relative caspase3/7 activity of glucose-deprived VCaP cells treated with either DMSO or 10 μM SIRT1 inhibitor for an indicated time were measured. ROS scavenger; Tiron (5 mM) was added for the last 12 h as indicated. Experiments were performed as biological triplicates (mean ± SD). For 3c–j one-way ANOVA with Dunnett’s multiple comparison test used for statistical significance were performed; where ****p < 0.0001, ***p = 0.0001, **p = 0.001, *p = 0.01, n.s not significant.

Interaction studies indicate that PGC1α deacetylation was crucial for the interaction and function of ERG. In this regard, SIRT1, an important regulator of PGC1α function under metabolic stress had been shown to deacetylate and activate PGC1α^29,33,35^. Deacetylated PGC1α becomes accessible for binding to various protein partners during metabolic stress^27,28^. Since inhibition of *SIRT1* has been reported to suppress prostate cancer growth^36,37^, we silenced SIRT1 in VCaP cells to interrogate its role in ERG-PGC1α interaction (Fig. S3a). As reported earlier^29^, we found that SIRT1 interacts and deacetylates PGC1α under glucose deprivation (Fig. 3a). We observed ERG interaction with PGC1α in the presence of SIRT1 which was absent in *SIRT1* silenced cells during glucose deprivation (Fig. 3a). SIRT1 specific inhibitor^38^; EX-527 also potentiated similar loss of interaction between ERG and PGC1α proteins under glucose stress (Fig. 3b). The SPP1-Luc assay showed no transactivation during glucose or serum starvation in the presence of an SIRT1 inhibitor (Figs. 3c and S3b). These observations indicate that SIRT1 deacetylates PGC1α under stress and promotes its coactivation function towards ERG. We further observed that stress-induced PGC1α could not increase ERG target genes expression during metabolic stress in the absence of *SIRT1* (Fig. S3c). Exogenous ERG expression that shall mimic strong ERG activation was able to restore SPP1-Luc activity in the presence of SIRT1 inhibitor confirming ERG transactivation as a downstream event (Fig. 3d). Together these results suggest that SIRT1 mediated deacetylation of PGC1α is necessary for the transactivation of ERG under metabolic stress.

### Physiological role of ERG- PGC1α axis during metabolic stress

Metabolic stress results in the generation of reactive oxygen species (ROS) that affect cell fate^39,40^. ROS clearance is an effective mechanism that prostate cancer tumors might utilize to overcome nutrient burden while acquiring castration resistance^41,42^. The role of ERG in regulating ROS is not well understood during PCa development. We observed increased ROS levels in ERG silenced VCaP cells (VCaP siERG) undergoing metabolic stress (Figs. 3e and S3d). Abrogation of PGC1α using shRNA or PGC1α-inhibitor SR18292 mediated a similar increase of ROS during metabolic stress (Figs. 3f and S3e, f). We manipulated SIRT1 using inhibitor; EX-527 and observed an increase in ROS levels of VCaP cells undergoing metabolic stress (Fig. 3g). We further verified if the increased ROS was a direct effect of SIRT1-PGC1α-ERG axis perturbations by performing rescue experiments using the PGC1α (1–400) mutant which was able to function similarly to full-length PGC1α in our interaction and transactivation assays. Interestingly, the PGC1α (1–400) domain was able to curtail ROS generation during glucose starvation in VCaP PGC1αKD cells but not in ERG silenced VCaP cells indicating PGC1 acts via ERG for ROS clearance (Fig. 3h). In the case of SIRT1 inhibition, exogenous ERG which shall mimic strong ERG activation (Fig. 3d) was able to completely reduce ROS generated during glucose stress (Fig. S3g). However, the PGC1α (1–400) domain could partially curtail ROS (~20% decrease) in cells with SIRT1 inhibition (Fig. S3g) suggesting SIRT1 regulates functional forms of PGC1 to some extent. We checked for apoptosis in VCaP cells undergoing energy stress. Concomitant with increased ROS, we observed increased caspase3/7 activity in VCaP cells where *ERG, PGC1*α, or *SIRT1* was abrogated using RNAi or inhibitors (Figs. 3i, j and S3h, i). ROS scavenger; tiron was able to neutralize oxidative stress and abrogate subsequent apoptosis, suggesting that apoptosis was due to increased levels of ROS (Figs. 3i, j and S3h, i). We envisage that ERG fusion-positive prostate cancer cells are susceptible to energy stress and the PGC1α-Sirt1 axis might provide survival benefits to tumors by activating antioxidant and metabolic functions of ERG fusion.

### PGC1α mediated transactivation of ERG fusion revealed antioxidant target genes

**Fig. 4:**
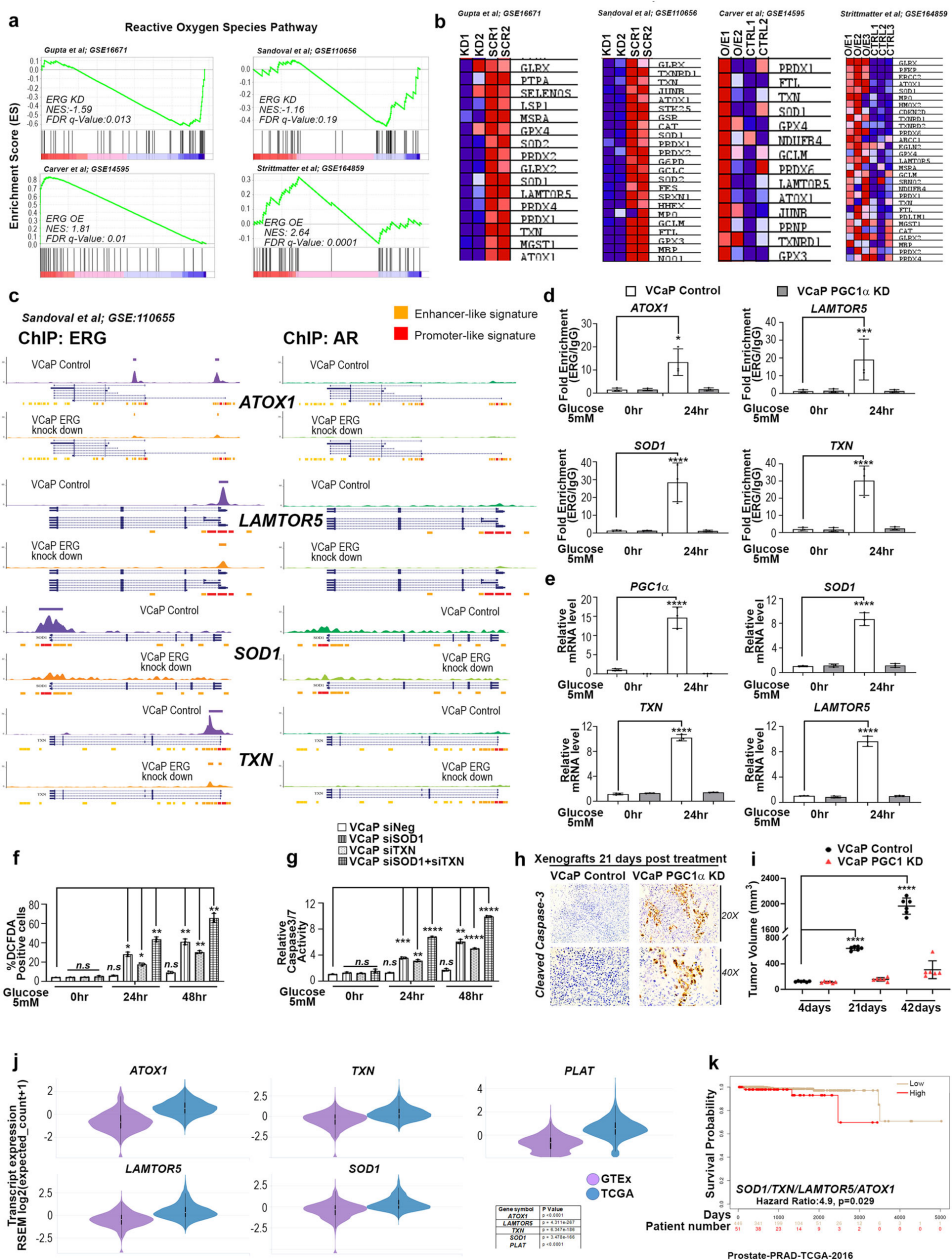
PGC1α mediated transactivation of ERG fusion revealed novel antioxidant target genes. **a** Enrichment plot from GSEA was conducted with datasets of ERG Knockdown (top panel) and ERG overexpression (bottom panel). Plots represent statistically significant enrichment (FDR <0.25) of “reactive oxygen species pathway” genes. **b** Heatmap of core enriched genes in “reactive oxygen species pathway” from GSEA analysis as indicated. **c** Aligned chromosomal peak regions and enriched ChIP signals (ERG ChIP left and AR ChIP right) in bigwig format were visualized using the UCSC browser for indicated samples. **d** ChIP assay performed with either ERG or control IgG antibody on glucose-deprived cells. The fold enrichment of co-precipitating DNA was determined by qPCR for indicated promoters. **e** VCaP control or VCAP PGC1α KD cells treated for 24 h. Relative transcript levels of indicated genes were analyzed using qRT-PCR. **f** ROS levels of treated VCaP cells were measured by flow cytometry after staining with the fluorescent dye DCFDA. **g** Relative caspase3/7 activity of treated VCaP cells were measured. **h** Representative cleaved-caspase3 staining (20X top, 40X bottom) in VCaP control and VCaP PGC1α KD xenograft following STF on day 21. **i** VCaP (control) and VCaP(PGC1α KD) xenograft tumor volume (mm^3^) following STF on days 4, 21, and 42. Lines indicate the mean ± SD (*n* = 6 per group). **j** Violin plots for transcript level in PRAD patient data set (TCGA targeted GTEx) were accessed using the Xena browser. Data were *Z*-score normalized and *p* value calculated using Welch’s *t*-test. **k** Kaplan–Meier curves for survival probability of TCGA PRAD data separated into high- and low-risk groups. Survival analysis was performed using Surv Express software. Experiments were performed as biological triplicates (mean ± SD). Two-way ANOVA with multiple comparison tests used for statistical significance of 4e. For 4 d, f, g, i one-way ANOVA with Dunnett’s multiple comparison test used for statistical significance were performed; where ****p < 0.0001, ***p = 0.0001, **p = 0.001, *p = 0.01, n.s not significant.

Since our data revealed increased ROS in cells upon ERG-PGC1α axis modulation, we were interested in ERG inducible target genes that might affect ROS clearance. In order to understand the metabolic signatures of ERG fusion-positive prostate cancer, we interrogated the transcriptome of VCaP cells having perturbations of ERG (GSE16671, GSE110656 ERG knockdown or GSE14595; GSE164859 ERG overexpression). Gene set enrichment analysis identified a cluster of antioxidant genes that showed negative enrichment upon knockdown of ERG (ERG KD) in VCaP cells (Fig. 4a Top panel). We analyzed the data set with overexpression of ERG (ERG OE) in fusion negative cells and found the same cluster of antioxidant genes belonging to the reactive oxygen species pathway to be upregulated (Fig. 4a Bottom panel). Next, we picked the significant genes which show core enrichment of ROS pathways in more than one ERG perturbation (Fig. 4b). ERG binding at promoter regions of these core enriched genes were verified by revalidating ERG ChIP Seq datasets (GSE110655) from VCaP cells with ERG knockdown. Interestingly we found four antioxidant genes; *TXN, SOD1, LAMTOR5*, and *ATOX1* with ERG binding signatures at their promoters which were abolished upon knockdown of ERG (Fig. 4c). We verified two other datasets for ERG ChIP (GSE14092 and GSE28950) in VCaP cells and found ERG binding at the promoters of the same genes (Fig. S4a). We did not observe any AR binding at promoters of these genes, indicating a possibility of AR independent induction of these targets by ERG (Figs. 4c and S4a). We further confirmed the presence of a previously reported ERG binding consensus motif in the promoters of these genes^43,44^ (Fig. S4b).

To understand how the ERG-PGC1α axis relates to these genes under metabolic stress, we performed chromatin immunoprecipitation studies on VCaP PGC1αKD cells undergoing glucose deprivation. We found a loss of ERG binding at promoters of these genes in the absence of PGC1α (Fig. 4d). We checked the expression of these genes under various metabolic stress. Interestingly, *TXN*, *SOD1*, and *LAMTOR5* were significantly upregulated in VCaP control cells during glucose deprivation. No significant changes were observed in the absence of PGC1α or ERG during glucose stress indicating functions to be mediated via the ERG-PGC1α complex (Figs. 4e and S4c). We checked for modulation of these ERG target genes under serum stress and found them to be induced by ERG in a PGC1α dependent manner (Fig. S4d). Together these data indicate that PGC1α might regulate direct transcriptional target genes of ERG during metabolic stress.

In order to access the functional importance of these antioxidant genes, we checked ROS levels and caspase3/7 activity of VCap cells undergoing glucose starvation by silencing *TXN* and *SOD1* genes selectively. We found that silencing of *TXN* or *SOD1* genes abrogated ROS clearance in VCaP cells under metabolic stress (Fig. 4f). We also observed increased caspase3/7 activity in cells under metabolic stress in absence of these genes (Fig. 4g). Simultaneous knockdown of *SOD1* and *TXN* further elevated ROS and Caspase activity under metabolic stress. We observed similar results for Caspase3/7 activity in ERG expressing COLO320 cells during glucose stress (Fig. S4e). Under basal/unstressed conditions, we did not observe any significant caspase activity associated with the silencing of these genes alone (Fig. S4f). To corroborate our finding in vivo, we used intermittent short-term fasting (STF) on PCa xenografts as described previously^45,46^. We had subjected SCID mice (NOD.Cg-Prkdc^scid^) (The Jackson Laboratory, Bar Harbor, USA) bearing VCaP control or VCaP PGC1α knockdown xenografts to STF, as described(details in supplementary methods) previously^46^. Although no significant changes in animal body weight subjected to STF was observed (Fig. S4g). We found that Caspase3/7 activity increased in PGC1α silenced xenografts as compared to control which correlated with decreased tumor growth observed in PGC1α silenced xenograft-containing animals (Fig. 4h, i). Additionally, the expression of ERG target genes *SOD1*, *TXN*, and *PLAT* went down in PGC1α silenced xenograft as compared to control animals under STF (Fig. S4h). Further tumor growth between VCaP control or VCaP PGC1α knockdown xenograft animals not subjected to STF were comparable, indicating that PGC1α’s tumor-promoting function is activated only under starvation resulting in ERG-mediated ROS clearance and anti-apoptotic functions in control animals but fails in absence of PGC1α (Figs. S4i and 4i). We conclude that PGC1α silenced animals fail to initiate an ERG-mediated antioxidant response to metabolic stress resulting in ROS-mediated apoptosis and decreased tumor growth. Our in vitro results during metabolic stress were further verified using an SIRT1 inhibitor on VCAP xenografts containing animals. We observed significant Caspase3/7 staining on xenografts of animals treated with a combination of STF and SIRT1 inhibitor but not with SIRT1 inhibitor alone indicating SIRT1-PGC1 axis activity only during metabolic stress (Fig. S4j). Together, our in vivo studies supports the role of SIRT1 mediated deacetylation of PGC1α resulting in the coactivation of ERG antioxidant and protumorigenic functions during metabolic stress. Interestingly, we found high expression of antioxidant genes; *SOD1*, *TXN*, *LAMTOR5*, and *ATOX1* in prostate cancer patients having higher ERG fusion expression (Figs. 4j and S4k). Multivariant survival analysis predicts poor survival and higher tumor recurrence of PCa patients in correlation with high expression of these antioxidant genes (Figs. 4k and S4l). In conclusion, our study identified the ERG-PGC1 axis which provides antioxidant functions to PCa cells undergoing metabolic perturbations.

## Discussion

Interestingly metabolic stress including glucose, glutamine, and serum deprivation has been reported to inhibit PCa growth via altering the redox balance of cells^47–50^. Categorically, carbohydrate restriction in mouse retarded PCa tumor growth indicates adaptive glucose metabolism^51,52^. However, in depth understanding of mechanisms maintaining redox balance thereby providing adaptive benefits under nutrient-deprived state for PCa is not well understood. Our study on metabolic stress and ERG fusion-driven antioxidant functions is supportive of PCa metabolic program. We report that PGC1α acts as a coactivator of ERG fusion during metabolic stress resulting in the activation of antioxidant target genes of ERG. SIRT1 mediated deacetylation of PGC1α is necessary for ERG and PGC1α interaction and prevention of ROS-dependent apoptosis. Given high SIRT1 levels have been associated with PCa initiation, progression, and metastasis, our study suggests that modulation of SIRT1 can be an important vulnerability^36,37,53^.

Our study in PCa is corollary to oncogenic roles of PGC1α coactivator in various cancers^21,32,54,55^. Earlier reports suggest that PGC1α supports PCa progression by activating the PGC1α-AR program under androgen stimulation^21,32^. Our results with AR antagonist flutamide didn’t affect PGC1α mediated transactivation of ERG fusion suggesting an AR independent mechanism under metabolic stress. Additionally, we observed a synergistic increase in ERG transcriptional activity upon androgen stimulation in presence of PGC1α but not in its absence; indicating PGC1α is necessary for transactivation of ERG fusion under metabolic stress. As reported by others, we anticipate prolonged androgen stimuli to activate the PGC1α-AR-dependent program which is absent in our studies and is in support of a low PGC1α state (basal levels) or AR low environment^21,22,32^. On the contrary, PGC1α has been recently shown to have tumor suppressor functions in PCa^22,23^. In this context, the transcription factor ERRα is reported to cooperate with PGC1α and suppress PCa metastasis^22,23^. Interestingly, Torrano et al. report low endogenous levels of PGC1α in basal cellular context while cautioning about nonbasal conditions like stress to alter expression levels; which might explain our contradictory observation with respect to induced endogenous levels of PGC1α under metabolic stress^22^. Opposingly, ERRα is reported to form a reciprocal regulatory loop with ERG fusion which can steer PCa progression^56^. We contemplate that the ERRα-PGC1α studies exogenously overexpress PGC1α in ERG fusion negative PC3 and DU145 cells thus unable to acknowledge ERG fusion’s regulation over ERRα^22,23,56^. PGC1α has been reported to alter promoter specificity of various transcription factors under nonbasal conditions of metabolic stress^27,28,30^. We envisage that PGC1α might alter promoter specificity of ERG during nonbasal conditions thus overriding ERRα function. Overall, our study highlights a prominent oncogenic role of the PGC1α-ERG axis during metabolic stress, where elevated PGC1α levels resulted in a pro-survival response.

In recent years, intermittent caloric restriction in combination with chemotherapy has gained importance and clinical trials are being proposed for cancers including PCa with dietary restrictions^57^ (NCT02126449, NCT04288336, NCT02710721, NCT04292041, and NCT03478904). The previous finding on dietary restrictions alone in PCa xenograft-containing mice had opposing results on survival and tumor growth^45,46^. Under short-term fasting, we observed tumor inhibition in mice bearing PGC1α silenced xenografts in comparison to ones having intact PGC1α - ERG axis. Our findings on intermittent caloric restrictions with abolished ERG-PGC1 axis might indicate a positive outcome for patients with low PGC1α levels.

Oxidative stress has been insinuated into PCa initiation, aggressiveness, castration resistance, and yet clinical interventions with antioxidant agents is not very promising^58–62^. Although antioxidant genes like *SOD3*, *TXN*, and *SOD1* has been associated with PCa aggressiveness and metastasis, none have shown a direct relationship with ERG fusion^63–65^. Our study identified the previously unknown antioxidant function of ERG fusion under metabolic stress which is mitigated through its direct transcriptional target genes *SOD1* and *TXN*. We speculate that PCa initiation under a nutrient-rich environment would show high ROS levels and cancer growth as reported previously^61,62^. While nutrient deficiency at later stages of the disease would activate the PGC1α-ERG axis for prolonged survival via ROS clearance.

*SOD1* and *TXN* has been previously shown to have a profound role in the progression of various cancers^66,67^. We observed that simultaneous inhibition of *SOD1* and *TXN* genes during metabolic stress caused significant apoptosis in PCa cells. Our study showed high expression levels of these genes in ERG fusion-positive PCa patients and their associated survival risk. Given the recurrence of ERG fusion in ~50% of patients, these newly found antioxidant functions might provide a therapeutic target for ERG positive Pca patients^2,7^. We surmise that PGC1α mediated coactivation of ERG fusion is largely tumor type and context-dependent and might be advantageous during the transition to castration-resistant phenotype.

## Methods

### Cell lines and culture conditions

We obtained VCaP (ATCC:CRL-2876™) from ATCC (Manassas, VA). PC3 and Colo320 cells was purchased from the National center for cell sciences (Pune, India). We cultured PC3 in RPMI medium (Thermo Fisher Scientific) and Colo320, VCaP cells in DMEM medium (Thermo Fisher Scientific). We supplemented the growth medium with 10% fetal bovine serum (Thermo Fisher Scientific), and cells were maintained at 37 °C in 5% CO_2_ for all experiments. The identity of all cell lines purchased were confirmed by STR analysis. All cell lines were determined to be mycoplasma free using the MycoAlert Plus system and kit (Lonza). For inducing glucose starvation, cells were grown in glucose-free DMEM (Thermo Fisher Scientific) containing dialyzed FBS (Thermo Fisher Scientific) and 5 mM glucose (Sigma) as described previously^30^. Control cells for starvation experiments were also grown in the same glucose-free DMEM containing dialyzed FBS and 25 mM glucose for basal conditions. For inducing serum starvation, cells were grown in serum-free DMEM (Thermo Fisher Scientific). For cold stress, cells were exposed to 4 °C as described previously^30^.

### Compounds, siRNAs, plasmids, and transfections

See Supplementary Table 1 for compounds and siRNAs used in this study. All compounds were dissolved in DMSO at room temperature except glucose and AICAR that were dissolved in Phosphate buffered saline. All siRNAs were transfected using RNAi-Max (Thermo Fisher Scientific). All Plasmids used are listed in Supplementary Table 2. The *SPP1* gene promoter with ERG binding site as described previously^31^; was cloned in the PGL4.24 luciferase reporter vector (Promega). PGC1alpha shRNAs were cloned in the pBABE-U6-shRNA plasmid (see Supplementary Table 2). pcDNA3FlagERG was a gift from Christopher Vakoc (Addgene plasmid # 66977). pcDNA-f:PGC1alpha, pcDNAflagPGC1alpha (180–797), and pcDNA-f:PGC1b were gifts from Bruce Spiegelman (Addgene plasmid # 1026, Addgene plasmid # 8943, Addgene plasmid # 1031). For transgene expression studies, plasmid DNA was transfected using Lipofectamine 3000 (Thermo Fisher Scientific). We used puromycin (2 µg/ml) to select for stably expressing cells (Thermo Fisher Scientific).

### Gene expression

For real-time PCR analysis, total RNA from cells and tumor tissues were extracted using an RNA extraction kit (Ambion, Life technologies) and cDNA synthesis using the iScript cDNA synthesis kit (Bio-Rad Laboratories) was performed following the manufacturer’s protocol. qPCR was performed using the primer sets listed in Supplementary Table 2. Relative gene expression was assessed using three biological replicas and data are shown as the mean ± SD. 18sRNA was used as an internal control for all samples.

### Immunoblotting and immunoprecipitation analysis

Whole-cell lysates from cells were prepared using lysis buffer (Thermo Fisher Scientific) supplemented with protease inhibitor and PhosphoSTOP tablets (Merck Millipore). An equal amount of total cellular proteins per sample was subjected to SDS–PAGE, transferred to a PVDF membrane (Merck Millipore) and probed with required antibodies. See Supplementary Table 3 for antibodies used in this study.

Immunoprecipitations were performed using 500 μg cell extracts as described previously^68^ and incubated with antibodies and precleared Protein A/G plus agarose (Santacruz Biotechnology). Subsequent immunoblots were performed as described above.

### Chromatin Immunoprecipitation analysis

Chromatin immunoprecipitations were performed using ChIP Kit (Millipore) according to the manufacturer’s instructions. Briefly, 1 × 10^7^ cells were fixed using 1% formaldehyde and then lysed using SDS lysis buffer. We incubated 500 µl of sonicated chromatin with either anti-ERG antibody or normal rabbit IgG antibody overnight at 4 °C followed by 2 h incubation in Agarose bead Slurry. Precipitated chromatin DNA cross-links were reversed by incubation at 65 °C for 4 h and immunoprecipitated DNA was analyzed by qPCR. We normalized values to those derived from normal IgG-precipitated. qPCR primer sequences for ChIP are listed in Supplementary Table 2. Fold-enrichment was assessed using three biological replicas of each treatment and data are shown as the mean ± SD.

### Functional assays

For luciferase assays, cells were treated as indicated 24 h post-transfection. Post experiment, luciferase activity was determined using the Dual Glo Luciferase Reporter assay system (Promega) as per the manufacturer’s instruction. The difference in transfection efficiency across samples was normalized by co-transfecting pRL-TK which expresses *Renilla* luciferase. Caspase activity was measured using Caspase Glo 3/7 assay system (Promega) per the manufacturer’s instruction. For all functional assays, three biological replicas were used and data were shown as mean ± SD.

ROS generation was measured using CH_2-_ DCFDA (Life Technologies). The cells were incubated with 10 mM DCFDA at 37 °C for 30 min. The cells were then harvested and resuspended in culture media. Samples were analyzed by flow cytometry (BD Biosciences) and data analyzed using Flo Jo software.

### Statistics and reproducibility

One-way analysis of variance (one-way ANOVA) was performed in Prism (GraphPad) using Dunnett’s multiple comparisons test to correct for multiple comparisons to the same control group; a *p* value < 0.05 was considered significant. Brown Forsythe Welch corrections were performed for variation in SD. Two-way analysis of variance (two-way ANOVA) was performed in Prism (GraphPad) using Sidak’s multiple comparisons test to correct for multiple comparisons to the same control group; a *p* value < 0.05 was considered significant. All experiments were performed in triplicate and mean ± SD was used to represent data.

### Reporting summary

Further information on research design is available in the Nature Research Reporting Summary linked to this article.

## Supplementary information

Supplementary Information

Description of Additional Supplementary Files

Supplementary Data 1

Supplementary Data 2

Reporting Summary

## Data Availability

The following datasets are used for GSEA analysis GSE16671, GSE110656, GSE14595; GSE164859. For ChIP-sequencing data analysis GSE110655, GSE14092, and GSE28950 were used. All datasets used for the study are listed in Supplementary Table 4. Details of methods and tools used are described in Supplementary Methods. All raw data used in the study are provided in Supplementary Data 1 and Supplementary Data 2. Other online tools like UCSC XENA browser used TCGA PRAD and GTex data, SURV express used TCGA prostate cancer and GSE40272 datasets.

## References

[1] Klezovitch O (2008). A causal role for ERG in neoplastic transformation of prostate epithelium. Proc. Natl Acad. Sci. USA.

[2] Mehra R (2008). Characterization of TMPRSS2-ETS gene aberrations in androgen-independent metastatic prostate cancer. Cancer Res..

[3] Tomlins SA (2008). Role of the TMPRSS2-ERG gene fusion in prostate cancer. Neoplasia.

[4] Shimizu K (1993). An ets-related gene, ERG, is rearranged in human myeloid leukemia with t(16;21) chromosomal translocation. Proc. Natl Acad. Sci. USA.

[5] Sorensen PH (1994). A second Ewing’s sarcoma translocation, t(21;22), fuses the EWS gene to another ETS-family transcription factor, ERG. Nat. Genet.

[6] Tomlins SA (2007). Distinct classes of chromosomal rearrangements create oncogenic ETS gene fusions in prostate cancer. Nature.

[7] Tomlins SA (2005). Recurrent fusion of TMPRSS2 and ETS transcription factor genes in prostate cancer. Science.

[8] Birdsey GM (2008). Transcription factor Erg regulates angiogenesis and endothelial apoptosis through VE-cadherin. Blood.

[9] Loughran SJ (2008). The transcription factor Erg is essential for definitive hematopoiesis and the function of adult hematopoietic stem cells. Nat. Immunol..

[10] Vijayaraj, P. et al. Erg is a crucial regulator of endocardial-mesenchymal transformation during cardiac valve morphogenesis. *Development* **139**, 3973–3985 (2012). 10.1242/dev.081596 PMC3472597 22932696

[11] Gopalan A (2009). TMPRSS2-ERG gene fusion is not associated with outcome in patients treated by prostatectomy. Cancer Res.

[12] Bluemn, E. G. et al. Androgen receptor pathway-independent prostate cancer is sustained through FGF signaling. *Cancer Cell* **32**, 474–489.e476 (2017). 10.1016/j.ccell.2017.09.003 PMC5750052 29017058

[13] Roudier, M. P. et al. Characterizing the molecular features of ERG-positive tumors in primary and castration resistant prostate cancer. *Prostate* **76**, 810–822 (2016). 10.1002/pros.23171 PMC5589183 26990456

[14] Shukla, S. et al. Aberrant activation of a gastrointestinal transcriptional circuit in prostate cancer mediates castration resistance. *Cancer Cell* **32**, 792–806.e797 (2017). 10.1016/j.ccell.2017.10.008 PMC5728174 29153843

[15] Bader, D. A. & McGuire, S. E. Tumour metabolism and its unique properties in prostate adenocarcinoma. *Nat. Rev. Urol.* **17**, 214–231 (2020). 10.1038/s41585-020-0288-x 32112053

[16] Reina-Campos, M. et al. Increased serine and one-carbon pathway metabolism by PKClambda/iota deficiency promotes neuroendocrineprostate cancer. *Cancer Cell* **35**, 385–400.e389 (2019). 10.1016/j.ccell.2019.01.018 PMC6424636 30827887

[17] Shao, Y. et al. Metabolomics and transcriptomics profiles reveal the dysregulation of the tricarboxylic acid cycle and related mechanisms in prostate cancer. *Int. J. Cancer* **143**, 396–407 (2018). 10.1002/ijc.31313 29441565

[18] Eidelman, E., Twum-Ampofo, J., Ansari, J. & Siddiqui, M. M. The metabolic phenotype of prostate cancer. *Front. Oncol.* **7**, 131 (2017). 10.3389/fonc.2017.00131 PMC5474672 28674679

[19] Hansen, A. F. et al. Presence of TMPRSS2-ERG is associated with alterations of the metabolic profile in human prostate cancer. *Oncotarget* **7**, 42071–42085 (2016). 10.18632/oncotarget.9817 PMC5173117 27276682

[20] Meller, S. et al. Integration of tissue metabolomics, transcriptomics and immunohistochemistry reveals ERG- and gleason score-specific metabolomic alterations in prostate cancer. *Oncotarget* **7**, 1421–1438 (2016). 10.18632/oncotarget.6370 PMC4811470 26623558

[21] Shiota, M. et al. Peroxisome proliferator-activated receptor gamma coactivator-1alpha interacts with the androgen receptor (AR) and promotes prostate cancer cell growth by activating the AR. *Mol. Endocrinol*. **24**, 114–127 (2010). 10.1210/me.2009-0302 PMC5428145 19884383

[22] Torrano, V. et al. The metabolic co-regulator PGC1alpha suppresses prostate cancer metastasis. *Nat. Cell Biol.* **18**, 645–656 (2016). 10.1038/ncb3357 PMC4884178 27214280

[23] Valcarcel-Jimenez, L. et al. PGC1alpha suppresses prostate cancer cell invasion through ERRalpha transcriptional control. *Cancer Res.* **79**, 6153–6165 (2019). 10.1158/0008-5472.CAN-19-1231 31594836

[24] Finck BN, Kelly DP (2006). PGC-1 coactivators: inducible regulators of energy metabolism in health and disease. J. Clin. Invest.

[25] Girnun, G. D. The diverse role of the PPARgamma coactivator 1 family of transcriptional coactivators in cancer. *Semin. Cell Dev. Biol*. **23**, 381–388 (2012). 10.1016/j.semcdb.2012.01.007 PMC3369022 22285815

[26] Luo, C., Widlund, H. R. & Puigserver, P. PGC-1 coactivators: shepherding the mitochondrial biogenesis of tumors. *Trends Cancer* **2**, 619–631 (2016). 10.1016/j.trecan.2016.09.006 PMC5465638 28607951

[27] Tan, Z. et al. The role of PGC1alpha in cancer metabolism and its therapeutic implications. *Mol. Cancer Ther*. **15**, 774–782 (2016). 10.1158/1535-7163.MCT-15-0621 27197257

[28] Lin J, Handschin C, Spiegelman BM (2005). Metabolic control through the PGC-1 family of transcription coactivators. Cell Metab..

[29] Canto C, Auwerx J (2009). PGC-1alpha, SIRT1 and AMPK, an energy sensing network that controls energy expenditure. Curr. Opin. Lipido..

[30] Sen, N., Satija, Y. K. & Das, S. PGC-1alpha, a key modulator of p53, promotes cell survival upon metabolic stress. *Mol. Cell* **44**, 621–634 (2011). 10.1016/j.molcel.2011.08.044 22099309

[31] Flajollet, S. et al. Abnormal expression of the ERG transcription factor in prostate cancer cells activates osteopontin. *Mol. Cancer Res.* **9**, 914–924 (2011). 10.1158/1541-7786.MCR-10-0537 21669963

[32] Tennakoon, J. B. et al. Androgens regulate prostate cancer cell growth via an AMPK-PGC-1alpha-mediated metabolic switch. *Oncogene* **33**, 5251–5261 (2014). 10.1038/onc.2013.463 PMC4009392 24186207

[33] Rodgers, J. T. et al. Nutrient control of glucose homeostasis through a complex of PGC-1alpha and SIRT1. *Nature* **434**, 113–118 (2005). 10.1038/nature03354 15744310

[34] Puigserver, P. et al. Activation of PPARgamma coactivator-1 through transcription factor docking. *Science* **286**, 1368–1371 (1999). 10.1126/science.286.5443.1368 10558993

[35] Rodgers, J. T. et al. Metabolic adaptations through the PGC-1 alpha and SIRT1 pathways. *FEBS Lett*. **582**, 46–53 (2008). 10.1016/j.febslet.2007.11.034 PMC2275806 18036349

[36] Byles, V. et al. SIRT1 induces EMT by cooperating with EMT transcription factors and enhances prostate cancer cell migration and metastasis. *Oncogene* **31**, 4619–4629 (2012). 10.1038/onc.2011.612 PMC4157820 22249256

[37] Huang, S. B. et al. Androgen deprivation-induced elevated nuclear SIRT1 promotes prostate tumor cell survival by reactivation of AR signaling. *Cancer Lett*. **505**, 24–36 (2021). 10.1016/j.canlet.2021.02.008 33617947

[38] Solomon JM (2006). Inhibition of SIRT1 catalytic activity increases p53 acetylation but does not alter cell survival following DNA damage. Mol. Cell Biol..

[39] Ahmad IM (2005). Mitochondrial O2*- and H2O2 mediate glucose deprivation-induced stress in human cancer cells. J. Biol. Chem..

[40] Aykin-Burns N, Ahmad IM, Zhu Y, Oberley LW, Spitz DR (2009). Increased levels of superoxide and H2O2 mediate the differential susceptibility of cancer cells versus normal cells to glucose deprivation. Biochem. J..

[41] Han, C. *et al*. Roles of reactive oxygen species in biological behaviors of prostate cancer. *Biomed. Res. Int.* **2020**, 1269624 (2020). 10.1155/2020/1269624 PMC7538255 33062666

[42] Luo, X. H. et al. KLF14 potentiates oxidative adaptation via modulating HO-1 signaling in castrate-resistant prostate cancer. *Endocr. Relat. Cancer* **26**, 181–195 (2019). 10.1530/ERC-18-0383 30400002

[43] Martens, J. H. et al. ERG and FLI1 binding sites demarcate targets for aberrant epigenetic regulation by AML1-ETO in acute myeloid leukemia. *Blood* **120**, 4038–4048 (2012). 10.1182/blood-2012-05-429050 PMC3496958 22983443

[44] Nhili, R. et al. Targeting the DNA-binding activity of the human ERG transcription factor using new heterocyclic dithiophene diamidines. *Nucleic Acids Res*. **41**, 125–138 (2013). 10.1093/nar/gks971 PMC3592449 23093599

[45] Bonorden MJ (2009). Intermittent calorie restriction delays prostate tumor detection and increases survival time in TRAMP mice. Nutr. Cancer.

[46] Thomas, J. A., II et al. Effect of intermittent fasting on prostate cancer tumor growth in a mouse model. *Prostate Cancer Prostatic Dis.* **13**, 350–355 (2010). 10.1038/pcan.2010.24 20733612

[47] Bueno De Paiva, L. et al. Effects of RhoA and RhoC upon the sensitivity of prostate cancer cells to glutamine deprivation. *Small GTPases* **12**, 20–26, 10.1080/21541248.2018.1546098. 10.1080/21541248.2018.1546098 PMC7781845 30449238

[48] Gonzalez-Menendez, P. et al. GLUT1 protects prostate cancer cells from glucose deprivation-induced oxidative stress. *Redox Biol.* **17**, 112–127 (2018). 10.1016/j.redox.2018.03.017 PMC6007175 29684818

[49] Thomas R, Kim MH (2008). HIF-1 alpha: a key survival factor for serum-deprived prostate cancer cells. Prostate.

[50] White, E. Z. et al. Serum deprivation initiates adaptation and survival to oxidative stress in prostate cancer cells. *Sci. Rep.* **10**, 12505 (2020). 10.1038/s41598-020-68668-x PMC7385110 32719369

[51] Caso, J. et al. The effect of carbohydrate restriction on prostate cancer tumor growth in a castrate mouse xenograft model. *Prostate* **73**, 449–454 (2013). 10.1002/pros.22586 PMC3594433 23038057

[52] Freedland SJ (2008). Carbohydrate restriction, prostate cancer growth, and the insulin-like growth factor axis. Prostate.

[53] Bader, D. A. et al. Mitochondrial pyruvate import is a metabolic vulnerability in androgen receptor-driven prostate cancer. *Nat. Metab.* **1**, 70–85 (2019). 10.1038/s42255-018-0002-y PMC6563330 31198906

[54] Sancho, P. et al. MYC/PGC-1alpha balance determines the metabolic phenotype and plasticity of pancreatic cancer stem cells. *Cell Metab.* **22**, 590–605 (2015). 10.1016/j.cmet.2015.08.015 26365176

[55] Vazquez, F. et al. PGC1alpha expression defines a subset of human melanoma tumors with increased mitochondrial capacity and resistance to oxidative stress. *Cancer Cell* **23**, 287–301 (2013). 10.1016/j.ccr.2012.11.020 PMC3708305 23416000

[56] Xu, Z. et al. Nuclear receptor ERRalpha and transcription factor ERG form a reciprocal loop in the regulation of TMPRSS2:ERG fusion gene in prostate cancer. *Oncogene* **37**, 6259–6274 (2018). 10.1038/s41388-018-0409-7 PMC6265259 30042415

[57] Palomar-Cros, A. et al. The association of nighttime fasting duration and prostate cancer risk: results from the multicase-control (MCC) study in Spain. *Nutrients* **13**, 2662 (2021). 10.3390/nu13082662 PMC8399976 34444822

[58] Chaiswing, L., Zhong, W., Liang, Y., Jones, D. P. & Oberley, T. D. Regulation of prostate cancer cell invasion by modulation of extra- and intracellular redox balance. *Free Radic. Biol. Med.* **52**, 452–461 (2012). 10.1016/j.freeradbiomed.2011.10.489 PMC3253260 22120495

[59] Gaziano JM (2009). Vitamins E and C in the prevention of prostate and total cancer in men: the Physicians’ Health Study II randomized controlled trial. JAMA.

[60] Klein, E. A. et al. Vitamin E and the risk of prostate cancer: the selenium and vitamin E cancer prevention trial (SELECT). *JAMA* **306**, 1549–1556 (2011). 10.1001/jama.2011.1437 PMC4169010 21990298

[61] Kumar B, Koul S, Khandrika L, Meacham RB, Koul HK (2008). Oxidative stress is inherent in prostate cancer cells and is required for aggressive phenotype. Cancer Res.

[62] Paschos, A., Pandya, R., Duivenvoorden, W. C. & Pinthus, J. H. Oxidative stress in prostate cancer: changing research concepts towards a novel paradigm for prevention and therapeutics. *Prostate Cancer Prostatic Dis* **16**, 217–225 (2013). 10.1038/pcan.2013.13 23670256

[63] Kim, J. et al. SOD3 acts as a tumor suppressor in PC-3 prostate cancer cells via hydrogen peroxide accumulation. *Anticancer Res.* **34**, 2821–2831 (2014). 24922645

[64] Vance, T. M. et al. Thioredoxin 1 in Prostate Tissue Is Associated with Gleason Score, Erythrocyte Antioxidant Enzyme Activity, and Dietary Antioxidants. *Prostate Cancer* **2015**, 728046, 10.1155/2015/728046. 10.1155/2015/728046 PMC4556330 26357575

[65] Young, B., Purcell, C., Kuang, Y. Q., Charette, N. & Dupre, D. J. Superoxide dismutase 1 regulation of CXCR4-mediated signaling in prostate cancer cells is dependent on cellular oxidative state. *Cell Physiol. Biochem.* **37**, 2071–2084 (2015). 10.1159/000438566 26599430

[66] Harris, I. S. et al. Glutathione and thioredoxin antioxidant pathways synergize to drive cancer initiation and progression. *Cancer Cell* **27**, 211–222 (2015). 10.1016/j.ccell.2014.11.019 25620030

[67] Papa, L., Manfredi, G. & Germain, D. SOD1, an unexpected novel target for cancer therapy. *Genes Cancer* **5**, 15–21 (2014). 10.18632/genesandcancer.4 PMC4063254 24955214

[68] Groisman R (2003). The ubiquitin ligase activity in the DDB2 and CSA complexes is differentially regulated by the COP9 signalosome in response to DNA damage. Cell.

