# Supplementary material for: PGC1 alpha coactivates ERG fusion to drive antioxidant target genes under metabolic stress
Source: Commun Biol. 2022 May 4;5:416. doi: 10.1038/s42003-022-03385-x (PMC9068611; doi:10.1038/s42003-022-03385-x)
Supplement: Supplementary file 1 — Supplementary Information [file 42003_2022_3385_MOESM1_ESM.pdf]

Title: PGC1 alpha coactivates ERG fusion to drive antioxidant target genes under metabolic stress.

Aiindrila Dhara[1], Imlimaong Aier[2], Ankush Paladhi[3], Pritish Kumar Varadwaj[2], Sumit Kumar Hira[3], Nirmalya Sen[1] ,*.

Affiliation: [1]Molecular oncology laboratory, S.N. Bose innovation centre, University of Kalyani, West Bengal 741235.
[2] Department of Bioinformatics & Applied Sciences, Indian Institute of Information Technology—Allahabad, Uttar Pradesh, India
[3] Cellular Immunology Laboratory, Department of Zoology, The University of Burdwan, Burdwan 713104, India
***** Corresponding: Nirmalya Sen, nirmalyarffs19@klyuniv.ac.in

Supplementary Information: File contains supplementary data figures, Figure legends, methods and unprocessed western blot images.

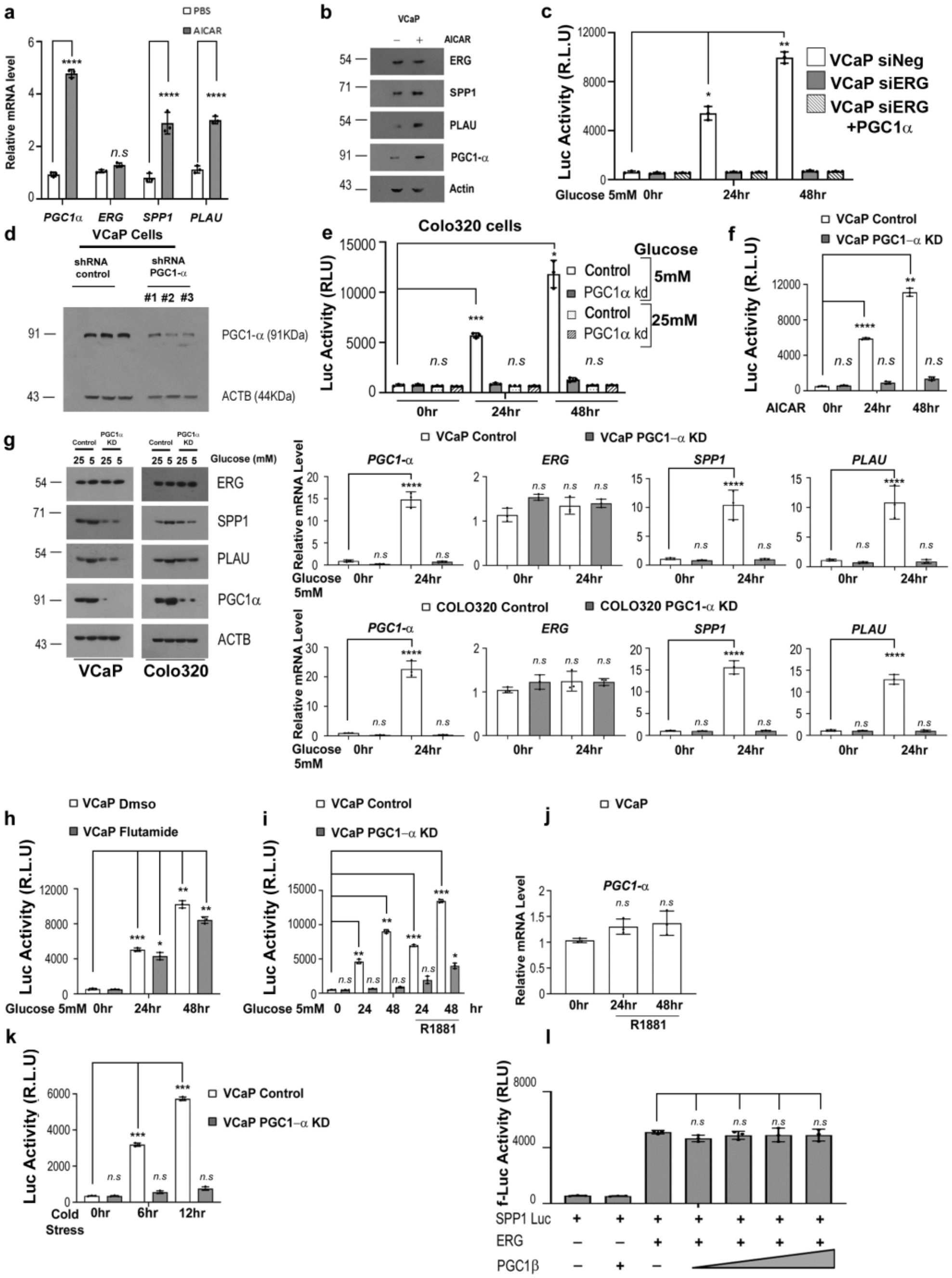

Figure S1: a) VCap Cells were treated with vehicle or AICAR (1mM) for 24 hours. Relative transcript levels of indicated genes were analyzed using qRT-PCR. **b)** VCaP cells were treated with vehicle or AICAR (1mM) for 24 hours and whole cell extract was immunoblotted using indicated antibodies. **c)** VCaP cells transfected with SPP1 luciferase alongside other constructs as indicated and were glucose deprived post transfection for indicated time. Luciferase assay was performed and relative firefly luciferase activity was plotted. **d)** Immunoblots of whole cell extract from VCaP (Scrambled shRNA) or VCAP PGC1α kd (PGC1α shRNA#1, #2, #3) stable cells using indicated antibodies. **e)** Colo320 control (Scrambled shRNA) or Colo320 PGC1α KD (PGC1α shRNA#1) stable cells transfected with SPP1 luciferase were glucose deprived(5mM) or cultured under basal condition(25mM) post transfection as indicated. Luciferase assay was performed and relative firefly luciferase activity was plotted. **f)** VCaP control or VCAP PGC1α KD stable cells

transfected with SPP1 luciferase were treated with vehicle or AICAR (1mM) post transfection as indicated. Luciferase assay was performed and relative firefly luciferase activity was plotted. **g)** (left panel) VCaP or COLO320 cells (control or PGC1α KD) stable cells were glucose starved as indicated for 24hours and whole cell extract was immunoblotted using indicated antibodies. (Right panel) VCaP or COLO320 cells were treated as indicated for 24 hours. Relative transcript levels of indicated genes were analyzed using qRT-PCR. **h)** VCaP Cells transfected with SPP1 luciferase were glucose deprived and treated with 0.1% DMSO or Flutamide (10μM) for indicated time. Luciferase assay was performed and relative firefly luciferase activity was plotted. **i)** VCaP control or VCAP PGC1α KD stable cells transfected with SPP1 luciferase were glucose deprived post transfection as indicated. Synthetic androgen; R1881(10nM) was added as indicated. Luciferase assay was performed and relative firefly luciferase activity was plotted. **j)** VCap Cells were treated with R1881(10nM) as indicated. Relative transcript levels of indicated genes were analyzed using qRT-PCR. **k)** VCaP control or VCAP PGC1α KD stable cells transfected with SPP1 luciferase received cold stress (4°C) post transfection as indicated. Luciferase assay was performed and relative firefly luciferase activity was plotted. **l)** PC3 cells were cotransfected as indicated. Luciferase assay was performed post 24 hours of transfection and relative firefly luciferase activity was plotted. Experiments were performed as biological triplicates (mean±SD). Two way ANOVA with multiple comparison test used for statistical significance of S1a, g. For S1c, e; f, h, i , j; k and l one way ANOVA with Dunnett's multiple comparison test used for statistical significance where ****p<.0001, ***p=0.0001,**p=0.001, *p=0.01, n.s=not significant

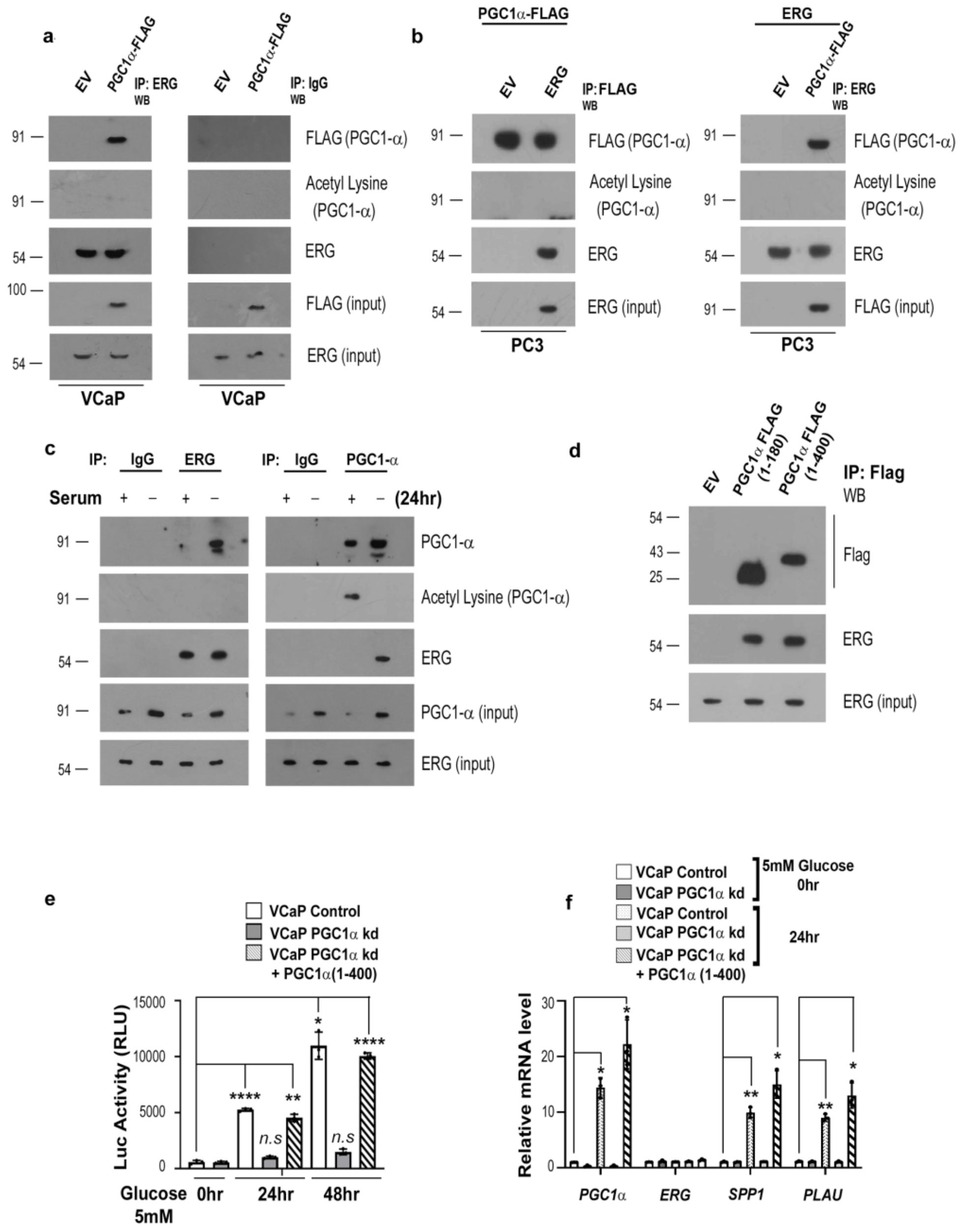

Figure S2 a) VCaP cells were transfected with either vector control (EV) or PGC1α Flag as indicated. Cells were harvested 24 hours post-transfection and subjected to immunoprecipitations using anti-ERG antibody or anti-IgG antibody, and western blots were performed for the indicated proteins. **b)** PC3 cells were transfected with either vector control (EV) or ERG or PGC1α Flag as indicated. Cells were harvested after 24 hours and subjected to immunoprecipitations using anti- FLAG antibody (Left) or anti-ERG antibody (Right), and western blots were performed for the indicated proteins. **c)** (Left panel) VCaP cells were treated as indicated. Cells were harvested after 24 hours and subjected to immunoprecipitations using anti- ERG antibody or anti-IgG antibody, and western blots

were performed for the indicated proteins. (Right panel) VCaP cells were treated as indicated. Cells were harvested after 24 hours and subjected to immunoprecipitations using anti- PGC1α antibody or anti-IgG antibody, and western blots were performed for the indicated proteins. **d)** VCaP Cells were transfected with either EV (empty vector) or PGC1α FLAG (1-180) or PGC1α FLAG (1-400) constructs as indicated. Cells were harvested after 24 hours and subjected to immunoprecipitations using anti- FLAG antibody, and western blots were performed for the indicated proteins. **e)** VCaP control or VCAP PGC1α KD stable cells transfected with SPP1 luciferase alongside PGC1α (1-400) construct and were glucose deprived post transfection as indicated. Luciferase assay was performed and relative firefly luciferase activity was plotted **f)** VCaP control or VCAP PGC1α KD stable cells were glucose deprived as indicated. PGC1 construct was transfected before removal of glucose as indicated. Relative transcript levels of indicated genes were analyzed using qRT-PCR. Experiments were performed as biological triplicates (mean±SD). Two way ANOVA with multiple comparison test used for statistical significance of S2f. For S2e one way ANOVA with Dunnett's multiple comparison test used for statistical significance where ****p<.0001, **p=0.001, *p=0.01, n.s=not significant

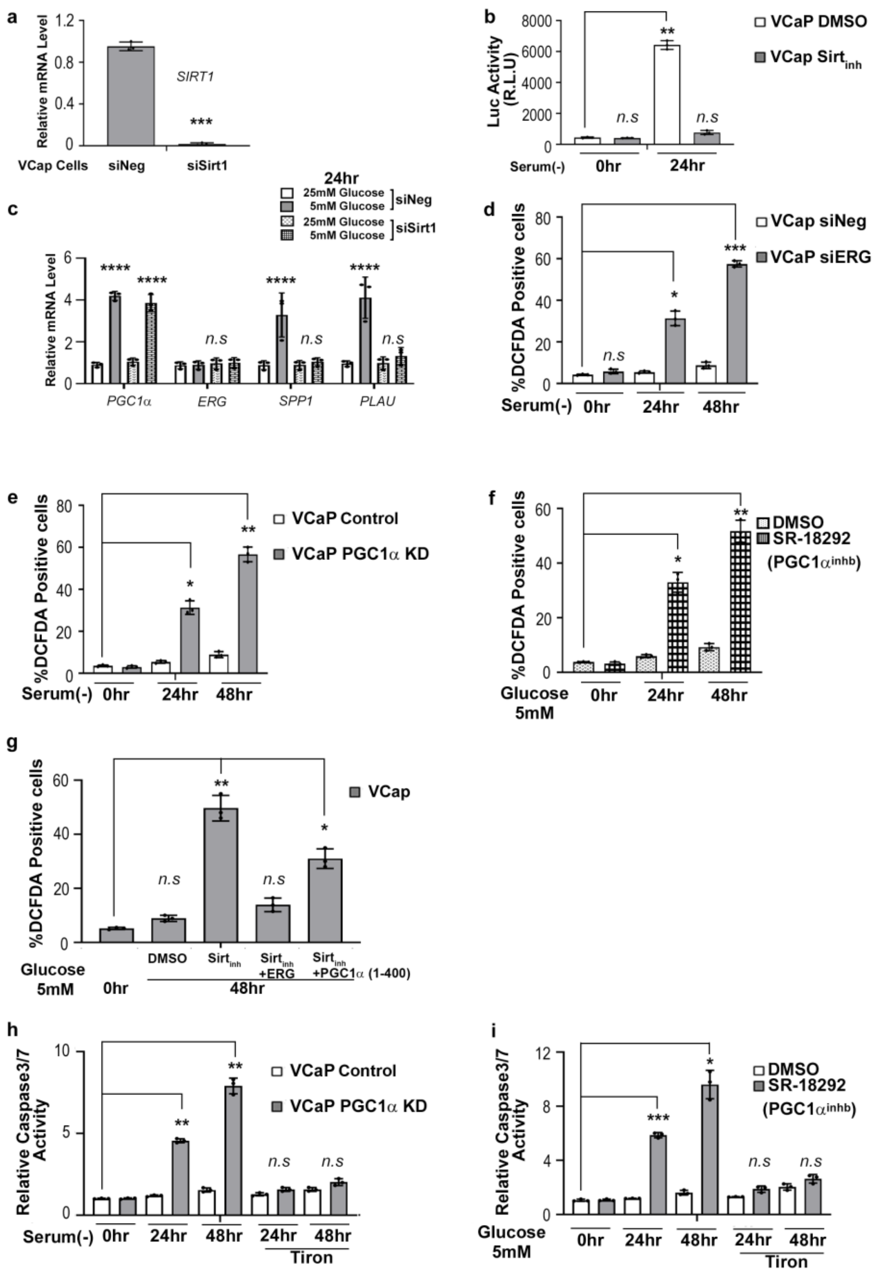

Figure S3 a) VCaP cells were transfected with either siRNA Negative control (SiNeg) or siRNA SIRT1. Relative transcript levels of indicated genes were analyzed using qRT-PCR; 48hours post transfection. Unpaired t-test with Welch's correction used for statistical significance where ***p=0.0001. **b)** SPP1 luciferase transfected VCaP cells were serum deprived and subjected to either 0.1%DMSO or 10μM of SIRT1 inhibitor as indicated. Luciferase assay was performed and relative firefly luciferase activity was plotted. **c)** Relative transcript levels of indicated genes were analyzed using qRT-PCR in glucose deprived SiRNA Negative control (SiNeg) or siRNA SIRT1 transfected VCaP cells. **d)** ROS levels in serum deprived VCaP control (VCaP siNeg) and ERG-knockdown cells (VCaP siERG) were measured by flowcytometry after staining with the fluorescent dye DCFDA. **e)** ROS levels in serum deprived VCaP control or VCAP PGC1α KD stable cells were measured by flowcytometry after staining with the fluorescent dye DCFDA. **f)** ROS levels in glucose deprived VCaP cells treated with either 0.1%DMSO or 10μM PGC1 α inhibitor was measured by flowcytometry after staining with the fluorescent dye DCFDA. **g)** VCaP cells were glucose deprived post transfection and subjected to either 0.1%DMSO or 10μM of SIRT1 inhibitor as indicated. ROS levels were measured by flowcytometry after staining with the fluorescent dye DCFDA. **h)** Relative

caspase 3/7 activity of serum deprived VCaP control or VCAP PGC1α KD stable cells were measured at indicated time. ROS scavenger; Tiron (5mM) was added for the last 10 hours as indicated. **i)** Relative caspase3/7 activity of glucose deprived VCaP cells treated with either DMSO or 10μM PGC1 α inhibitor for indicated time were measured. ROS scavenger; Tiron (5mM) was added for last 10 hours as indicated. Experiments were performed as biological triplicates (mean±SD). Unpaired t-test with welch's correction for statistical significance for S3A. Two way ANOVA with multiple comparison test used for statistical significance of S3c. For S3b,d e ;f ,g ,h and i one way ANOVA with Dunnett's multiple comparison test used for statistical significance where ****p<.0001, ***p=0.0001,**p=0.001, *p=0.01, n.s=not significant

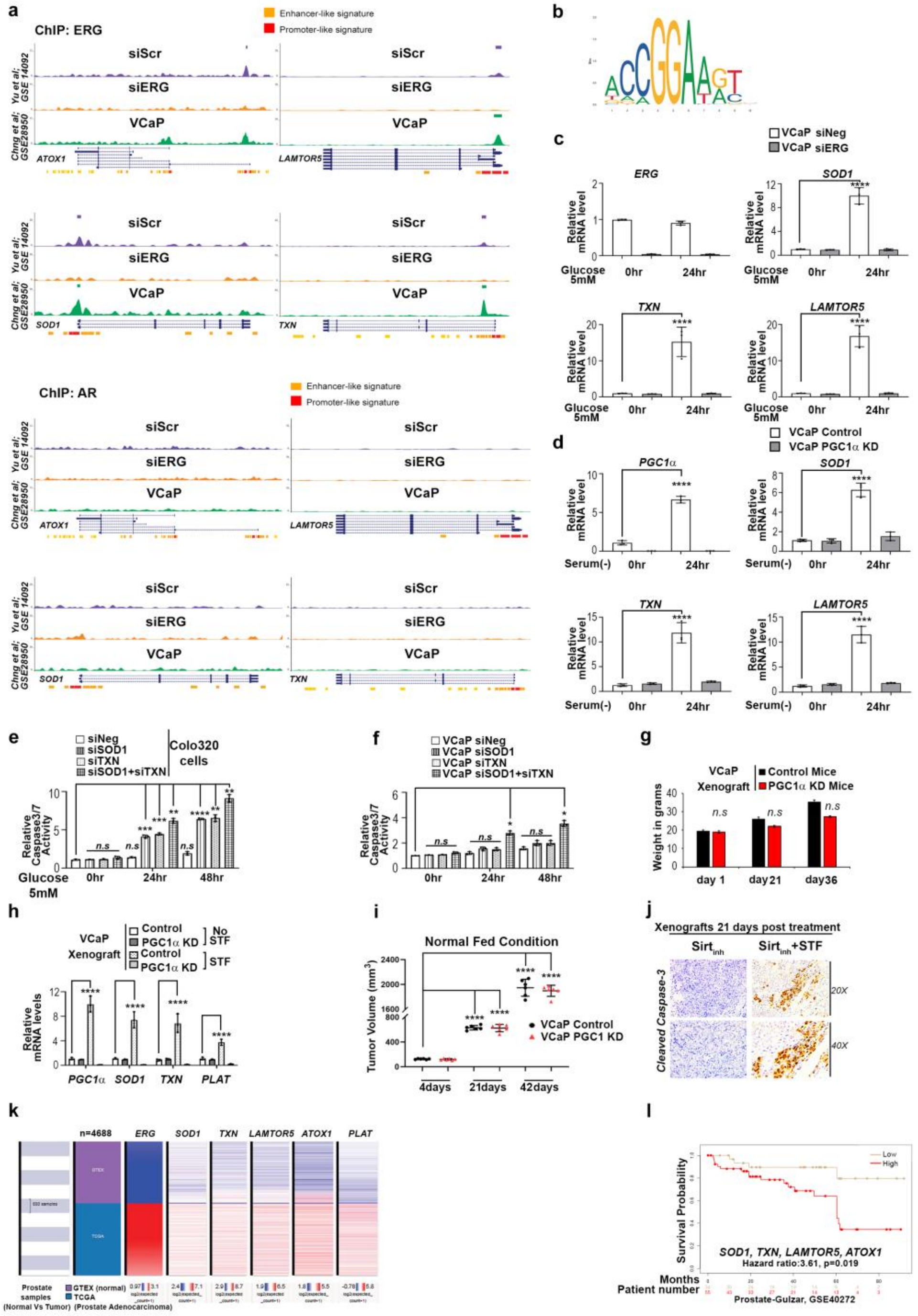

Figure S4 a) Aligned chromosomal peak regions and enriched ChIP signals (ERG ChIP top, AR ChIP bottom) in bigwig format for indicated genes were visualized using the UCSC browser for VCaP cells, Scrambles siRNA control(siScr) and ERG siRNA (siERG) samples. Cis-regulatory elements (cCREs) were depicted using the ENCODE data present in the browser. **b)** Depiction of transcription factor binding profile sequence logo for ERG in vertebrates as provided in JASPAR. **c)** Relative transcript levels of indicated genes were analyzed using qRT-PCR in glucose deprived VCaP control (VCaP siNeg) and ERG-knockdown (VCaP siERG) cells. **d)** Relative transcript levels of indicated genes were analyzed using qRT-PCR in glucose deprived VCaP control or VCAP PGC1α KD stable cells. **e)** Relative caspase3/7 activity of siRNA transfected Colo320 cells subjected to glucose deprivation as indicated were measured. **f)** Relative caspase3/7 activity of indicated siRNA transfected VCaP cells under basal conditions were measured. **g)** Body weight comparison for VCaP control or VCAP PGC1α KD xenograft containing mice on indicated days. Bars indicate the mean ± SD (n=6 per group). **h)** Relative transcript levels of indicated genes were analyzed using qRT-PCR from VCaP control or VCAP PGC1α KD xenograft on day 21 with or without STF.Animals used n=3 per groups. **i)** VCaP (control) and VCaP(PGC1α KD) xenograft tumor

volume (mm^3) following normal feed on day 4, 21 and 42. Lines indicate the mean ± SD (n=6 per group). **j)** Representative cleaved-caspase 3 staining (20X top, 40X bottom) in VCaP control xenograft following either SIRT1 inhibitor alone or in combination with STF on day 21. **k)** Heatmap(log2_expected count+1) of transcript level expression for indicated genes in prostate GTex normal and TCGA tumor data refined by ERG status using XENA browser. **l)** Kaplan-Meier curves for survival probability of PCa patients separated into high- and low-risk groups for the expression of the genes indicated in these two groups. Gulzar et al data set GSE40272 was used. Survival analysis was performed using Surv Express software. Experiments were performed as biological triplicates (mean±SD). Two way ANOVA with multiple comparison test used for statistical significance of S4c, d, h. For S3e, f, g, i; one way ANOVA with Dunnett's multiple comparison test used for statistical significance where ****p<.0001, ***p=0.0001,**p=0.001, *p=0.01, n.s=not significant

Supplementary methods:

Xenograft studies: All animal experimentation was reviewed and approved by RGCB, Institutional Animal Ethics Committee. SCID mice NOD.Cg-Prkdc^{scid} (The Jackson Laboratory) strain, all Male, were obtained from breeding colony maintained by RGCB animal facility and were handled in pathogen-free conditions. Xenograft formation was conducted using mice of 4-6 weeks of age injected subcutaneously with $1x10^6$ of VCaP cells (Control or PGC1α KD) in 200 µL of Matrigel (BD Biosciences) above the right flank[(1-3)]. Animals were euthanized when tumors approached the maximum accepted size (>2 cm in one dimension). Investigators were not blinded to the treatments used. To assess tumor regression, once tumors were ~350 mm^3, 12 mice per group were randomized (no selection based on tumor volume) for short term starvation as described previously [(4-6)]. STF was carried out 2 days per week for 24 hours with access to water. Two mice per group were sacrificed after 21 days for histological analysis of the tumors and three mice per group for transcript analysis. 5 mice per group were fed normal diet without STF and three mice were sacrificed after 21 days from each group for transcript analysis. For normally fed studies, 8 mice per group (VCaP Control or PGC1α KD) were randomized and tumors were allowed to grow till they reach endpoint of experiments. Only VCaP control xenograft mice were taken (6 mice per group; SIRT1inhibitor only/Inhibitor+STF groups were randomized) for SIRT1 inhibitor studies, EX-527 was injected at 10mg/Kg body weight on 3 days per week as described previously[(41,42)]. We observed the tumor volumes and body weights of the cohorts for 48 days at which point tumor sizes reached the maximum permissible limits within the protocol. Tumor volume was calculated using formula$(\pi \times D \times d^2)/6$

For histological analysis, we fixed xenograft tumor samples in formalin, and we processed the wet tissue into tissue sections using standard methods. All slides were imaged using Nikon 80i research microscope (200 & 400×). We assessed apoptosis by immunohistochemistry of cleaved caspase-3 (CC3) antibody.

Bioinformatics analysis: All data sets used are listed in **supplementary table 4**. Gene Set Enrichment Analysis was performed using program GSEA version 4.1.0 (http://www.broadinstitute.org/gsea/index.jsp) (7). Following ERG datasets were used in this study (GSE16671, GSE110656, GSE164859, GSE14595). The RNA-Seq sequences were aligned using HISAT2(8) against human reference genome hg38. The reads generated were then quantified using feature Counts (9), an efficient algorithm for assigning sequence reads to genomic features, to account for overlapping reads using the hg38 genomic region

reference file downloaded from GENCODE (10). The files generated were then subjected to normalization using DESeq2 (11) to make the expression level comparison more suitable for subsequent analysis using GSEA. Hallmark geneset from molecular signature database (MSigDB v5.1; http://software.broadinstitute.org/gsea/msigdb) was used with a permutation of 1000. Weighted enrichment statistics and meandiv normalization mode was used for all analysis. Pathways showing Normalized Enrichment Scores with FDR q value <0.25 was considered significant.

ChIP-Seq data were obtained from NCBI GEO database (12), the details for which are provided below (Table4). The sequences were aligned using Bowtie2 (13) against human reference genome hg38. Peak calling on the aligned sequences were performed using MACS2 (14) for narrow peaks with a minimum FDR (q-value) of 0.05 in order to identify transcription factor binding regions. The resultant data generated was converted to bigwig format using the Wig/BedGraph-to-bigWig converter tool present in Galaxy(15). The data was then visualized using the UCSC genome browser (16)using custom tracks. Depiction of transcription factor binding profile sequence logo for ERG in vertebrates as provided in JASPAR (17,18).

For transcript level expression in prostate cancer patients, we used https://xena.ucsc.edu/ platform (19). We choose TCGA target GTEx cohort for prostate adenocarcinoma and refined samples as ERG low (GTEx) and ERG high(TCGA) in phenotype(n=4688). RSEM expected counts of RNA seq data for selected gene transcripts were analyzed with respect to refined samples and average expression was plotted as heatmap(log2_expected count+1). For individual violin plots of transcript level expression, subgroups were compared as per study (TCGA target GTEx) and Z-score normalized. P value was calculated using welch's t-test.

For multivariant survival analysis, survexpress platform (20) was used. Analysis for genes were performed over PRAD-TCGA data set 2016 (21) and Gulzar-prostate data(22) (GSE40272) sets by averaging all probe sets per sample and quantile normalized data used. Risk group were maximized for censored survival or recurrence tumor data.

Supplementary tables:

Supplementary Table 1:

Targeting GENE	Company/Catalogue

Sirt1 (siRNA)	SCBT# 40986
TXN (siRNA)	SCBT# 106984
SOD1 (siRNA)	SCBT# 36523
All star Negative control (siRNA)	Qiagen# SI03650318
ERG (siRNA),	PMID: 24591637
**Chemical**	**Catalogue**
AICAR	TOCRIS# 2840
Glucose	SIGMA# G7021
Flutamide	TOCRIS# 4094
R1881	SIGMA# R0908
EX-527	TOCRIS# 2780
SR18292	Selleckchem# S8528
Tiron	SIGMA # D7389
Protein A/G plus agarose	SCBT# SC2003
DMSO	Himedia# TC185
Carboxy H2-DCFDA	Life technologies# C400
puromycin	Thermo Fisher Scientific# A1113803
Lipofectamine 3000	Thermo Fisher Scientific #L3000001
RNA Isolation Kit	Thermo Fisher Scientific# 12183018A
Caspase Glo 3/7 Promega	Promega# G8091
Dual Glo luciferase Assay system	Promega# E2920
EZ-ChIP Assay kit	MerckMillipore# 17-371
iScript cDNA synthesis kit	Biorad #1708891
iTaq universal syBRgreen supermix	Biorad# 1725121
Cell extraction Buffer	Thermo Fisher Scientific #FNN0011

Supplementary Table 2:

Gene	Forward	Reverse	Reference
**Real time PCR Primers**			
TMPRSS2-ERG	CGCGAGCTAAGCAGGAGGCG	CATGTTTGGGGGTGGCATGT	(23)
PGC1α	AACAGCAGCAGAGACAAATGCACC	TGCAGTTCCAGAGAGTTCCACACT	(24)
SPP1	TTCTGATTGGGACAGCCGTG	TCTCATCATTGGCTTTCCGCT-	(25)
PLAU			Qiagen; QT00013426
ATOX1			Qiagen;QT330001

LAMTOR5	ATGGAGCCAGGTGCAGGTC	TGGAGGGATTCTTCATTGTG	(26)
SOD1	AAGGCCGTGTGCGTGCTGAA	GGCCCACCGTGTTTTCTGGA	(27)
TXN	GATCAAGCCTTTCTTTCATTCCC	CCCACCTTTTGTCCCTTCTTAA	(28)
**ChIP qPCR Primers**			
ATOX1 (ChIP)	CAGAAGCCGCAGGCTGCA	ACAAGGTGGGGCCCGTGT	In this paper, Synthesized from Sigma
LAMTOR5 (ChIP)	TGGACCGTAACGGCGCCT	GGAATAATCCGTGCCGGC	In this paper, Synthesized from Sigma
SOD1 (ChIP)	GCGGAGGTCTGGCCTATAA	TGATGATGCCCTGCACTGG	In this paper, Synthesized from Sigma
TXN (ChIP)	ACGCTGGGCACCGCCACT	AAGGCGGTGGCGGGAGGT	In this paper
**PLASMID CONSTRUCTS**	**Backbone**	**ID/Sequence/source**	**Reference**
ERG	pcDNA3	Addgene# 66977	(29)
PGC1α FLAG	pcDNA3	Addgene plasmid # 1026	(30)
∆N-PGC1α FLAG	pcDNA3	Addgene plasmid # 8943,	(31)
PGC1β	pcDNA3	Addgene plasmid # 1031	(32)
PCDNA3 EV		Invitrogen	
SPP1 Luc (promoter)	PGL4.24	Promega	In This paper
PGC1a shRNA	pBABE-U6	GCAGAGTATGACGATGGTATT	(33)
		CCGTTATACCTGTGATGCTTT	(33)
		GAGCAAGTATGACTCTCTG	(34)
		GGGCTATCTGTCAGCTAAA	

Supplementary Table 3:

Antibody	Company	Catalogue
PGC1a	Thermofisher	PA572948
Spp1	Abcam	Ab181440
PLAU	Abcam	Ab133563
OctA-probe(G-8)/ FLAG	Santa cruz biotechnology	sc166384
ERG	abcam	Ab133264
Acetyl Lysine	Cell Signaling Technology	9441
Sirt1	abcam	ab110304
Anti rabbit	BioRad	170-6515
Anti mouse	Cell Signaling Technology	7076S
Beta-actin	ThermoFisher	MA5-15739
Normal Rabbit IgG	abcam	Ab171870
Cleaved caspase-3 cleavage (CC3)	Cell Signaling Technology	9661

Supplementary table 4

GEO ID	GSM	Sample name	STUDY
GSE110655	GSM3223706	VCaP shERG ERG	(35)
	GSM3223711	VCaP shCt AR	
	GSM3223712	VCaP shERG AR	
	GSM3323705	VCaP shCt ERG	
	GSM3223713	VCaP shCt Input	
	GSM3223714	VCaP shERG Input	
GSE14092	GSM353636	VCaP_NT_AR	(36)
	GSM353637	VCaP_NT_ERG	
	GSM353638	VCaP_siERG_AR	
	GSM353639	VCaP_siERG_ERG	
GSE28950	GSM717392	AR ChIP sequencing in VCaP cells	(37)
	GSM717395	ERG ChIP sequencing in VCaP cells	
	GSM717391	VCaP Genomic Input	
**GSE16671**	**GSM416785**	scramble shRNA in VCAP cells	(38)
	GSM16494	scramble shRNA in VCAP cells	
	GSM16495	ERG shRNA in VCAP cells	

	GSM16496	ERG shRNA in VCAP cells	
**GSE14595**	GSM364907	ERG O/E in 293 HEK cells	(39)
	GSM364908	ERG O/E in 293HEK cells	
	GSM364909	vector control in 293HEK cells	
	GSM3649010	vector control in 293HEK cells duplicate	
**GSE164859**	GSM5021156	RWPE1-Vector RNA-Seq Replicate 1	(40)
	GSM5021157	RWPE1-Vector RNA-Seq Replicate 2	
	GSM5021158	RWPE1-Vector RNA-Seq Replicate 3	
	GSM5021159	RWPE1-ERG O/ERNA-Seq Replicate 1	
	GSM5021160	RWPE1-ERG O/ERNA-Seq Replicate 2	
	GSM5021161	RWPE1-ERG O/ERNA-Seq Replicate 3	
**GSE110656**	GSM3005042	VCaP shCt 1	(35)
	GSM3005043	VCaP shCt 2	
	GSM3005044	VCaP shERG 1	
	GSM3005045	VCaP shERG 2	

Supplementary References:

1. Eswaraka J, Giddabasappa A, Han G, Lalwani K, Eisele K, Feng Z*, et al.* Axitinib and crizotinib combination therapy inhibits bone loss in a mouse model of castration resistant prostate cancer. BMC Cancer;14:742
2. Hargrove AE, Martinez TF, Hare AA, Kurmis AA, Phillips JW, Sud S*, et al.* Tumor Repression of VCaP Xenografts by a Pyrrole-Imidazole Polyamide. PLoS One;10:e0143161
3. Huhtaniemi R, Oksala R, Knuuttila M, Mehmood A, Aho E, Laajala TD*, et al.* Adrenals Contribute to Growth of Castration-Resistant VCaP Prostate Cancer Xenografts. Am J Pathol;188:2890-901
4. Buschemeyer WC, 3rd, Klink JC, Mavropoulos JC, Poulton SH, Demark-Wahnefried W, Hursting SD*, et al.* Effect of intermittent fasting with or without caloric restriction on prostate cancer growth and survival in SCID mice. Prostate;70:1037-43
5. Lee C, Raffaghello L, Brandhorst S, Safdie FM, Bianchi G, Martin-Montalvo A*, et al.* Fasting cycles retard growth of tumors and sensitize a range of cancer cell types to chemotherapy. Sci Transl Med;4:124ra27
6. Thomas JA, 2nd, Antonelli JA, Lloyd JC, Masko EM, Poulton SH, Phillips TE*, et al.* Effect of intermittent fasting on prostate cancer tumor growth in a mouse model. Prostate Cancer Prostatic Dis;13:350-5
7. Subramanian A, Tamayo P, Mootha VK, Mukherjee S, Ebert BL, Gillette MA*, et al.* Gene set enrichment analysis: a knowledge-based approach for interpreting genome-wide expression profiles. Proc Natl Acad Sci U S A 2005;102:15545-50
8. Kim D, Paggi JM, Park C, Bennett C, Salzberg SL. Graph-based genome alignment and genotyping with HISAT2 and HISAT-genotype. Nat Biotechnol 2019;37:907-15
9. Liao Y, Smyth GK, Shi W. featureCounts: an efficient general purpose program for assigning sequence reads to genomic features. Bioinformatics 2014;30:923-30
10. Frankish A, Diekhans M, Ferreira AM, Johnson R, Jungreis I, Loveland J*, et al.* GENCODE reference annotation for the human and mouse genomes. Nucleic Acids Res 2019;47:D766-D73
11. Love MI, Huber W, Anders S. Moderated estimation of fold change and dispersion for RNA-seq data with DESeq2. Genome Biol 2014;15:550
12. Barrett T, Wilhite SE, Ledoux P, Evangelista C, Kim IF, Tomashevsky M*, et al.* NCBI GEO: archive for functional genomics data sets--update. Nucleic Acids Res;41:D991-5
13. Langmead B, Salzberg SL. Fast gapped-read alignment with Bowtie 2. Nat Methods;9:357-9
14. Zhang Y, Liu T, Meyer CA, Eeckhoute J, Johnson DS, Bernstein BE*, et al.* Model-based analysis of ChIP-Seq (MACS). Genome Biol 2008;9:R137
15. Afgan E, Baker D, Batut B, van den Beek M, Bouvier D, Cech M*, et al.* The Galaxy platform for accessible, reproducible and collaborative biomedical analyses: 2018 update. Nucleic Acids Res;46:W537-W44
16. Kent WJ, Sugnet CW, Furey TS, Roskin KM, Pringle TH, Zahler AM*, et al.* The human genome browser at UCSC. Genome Res 2002;12:996-1006
17. Fornes O, Castro-Mondragon JA, Khan A, van der Lee R, Zhang X, Richmond PA*, et al.* JASPAR 2020: update of the open-access database of transcription factor binding profiles. Nucleic Acids Res;48:D87-D92
18. Stormo GD. Modeling the specificity of protein-DNA interactions. Quant Biol;1:115-30
19. Goldman MJ, Craft B, Hastie M, Repecka K, McDade F, Kamath A*, et al.* Visualizing and interpreting cancer genomics data via the Xena platform. Nat Biotechnol;38:675-8
20. Aguirre-Gamboa R, Gomez-Rueda H, Martinez-Ledesma E, Martinez-Torteya A, Chacolla-Huaringa R, Rodriguez-Barrientos A*, et al.* SurvExpress: an online biomarker validation tool and database for cancer gene expression data using survival analysis. PLoS One;8:e74250

21. The Molecular Taxonomy of Primary Prostate Cancer. Cell;163:1011-25
22. Gulzar ZG, McKenney JK, Brooks JD. Increased expression of NuSAP in recurrent prostate cancer is mediated by E2F1. Oncogene;32:70-7
23. Lu B, Maqsodi B, Yang W, McMaster GK, Perner S, Regan M*, et al.* Detection of TMPRSS2-ERG fusion gene expression in prostate cancer specimens by a novel assay using branched DNA. Urology 2009;74:1156-61
24. Bhalla K, Hwang BJ, Dewi RE, Ou L, Twaddel W, Fang HB*, et al.* PGC1alpha promotes tumor growth by inducing gene expression programs supporting lipogenesis. Cancer Res;71:6888-98
25. Zou B, Li J, Xu K, Liu JL, Yuan DY, Meng Z*, et al.* Identification of key candidate genes and pathways in oral squamous cell carcinoma by integrated Bioinformatics analysis. Exp Ther Med;17:4089-99
26. Liu F, You X, Wang Y, Liu Q, Liu Y, Zhang S*, et al.* The oncoprotein HBXIP enhances angiogenesis and growth of breast cancer through modulating FGF8 and VEGF. Carcinogenesis;35:1144-53
27. Li X, Chen Y, Zhao J, Shi J, Wang M, Qiu S*, et al.* The Specific Inhibition of SOD1 Selectively Promotes Apoptosis of Cancer Cells via Regulation of the ROS Signaling Network. Oxid Med Cell Longev;2019:9706792
28. Rodriguez-Garcia A, Hevia D, Mayo JC, Gonzalez-Menendez P, Coppo L, Lu J*, et al.* Thioredoxin 1 modulates apoptosis induced by bioactive compounds in prostate cancer cells. Redox Biol;12:634-47
29. Roe JS, Mercan F, Rivera K, Pappin DJ, Vakoc CR. BET Bromodomain Inhibition Suppresses the Function of Hematopoietic Transcription Factors in Acute Myeloid Leukemia. Mol Cell;58:1028-39
30. Monsalve M, Wu Z, Adelmant G, Puigserver P, Fan M, Spiegelman BM. Direct coupling of transcription and mRNA processing through the thermogenic coactivator PGC-1. Mol Cell 2000;6:307-16
31. Fan M, Rhee J, St-Pierre J, Handschin C, Puigserver P, Lin J*, et al.* Suppression of mitochondrial respiration through recruitment of p160 myb binding protein to PGC-1alpha: modulation by p38 MAPK. Genes Dev 2004;18:278-89
32. Lin J, Puigserver P, Donovan J, Tarr P, Spiegelman BM. Peroxisome proliferator-activated receptor gamma coactivator 1beta (PGC-1beta), a novel PGC-1-related transcription coactivator associated with host cell factor. J Biol Chem 2002;277:1645-8
33. Vazquez F, Lim JH, Chim H, Bhalla K, Girnun G, Pierce K*, et al.* PGC1alpha expression defines a subset of human melanoma tumors with increased mitochondrial capacity and resistance to oxidative stress. Cancer Cell;23:287-301
34. Sen N, Satija YK, Das S. PGC-1alpha, a key modulator of p53, promotes cell survival upon metabolic stress. Mol Cell;44:621-34
35. Sandoval GJ, Pulice JL, Pakula H, Schenone M, Takeda DY, Pop M*, et al.* Binding of TMPRSS2-ERG to BAF Chromatin Remodeling Complexes Mediates Prostate Oncogenesis. Mol Cell;71:554-66 e7
36. Yu J, Mani RS, Cao Q, Brenner CJ, Cao X, Wang X*, et al.* An integrated network of androgen receptor, polycomb, and TMPRSS2-ERG gene fusions in prostate cancer progression. Cancer Cell;17:443-54
37. Chng KR, Chang CW, Tan SK, Yang C, Hong SZ, Sng NY*, et al.* A transcriptional repressor co-regulatory network governing androgen response in prostate cancers. EMBO J;31:2810-23
38. Gupta S, Iljin K, Sara H, Mpindi JP, Mirtti T, Vainio P*, et al.* FZD4 as a mediator of ERG oncogene-induced WNT signaling and epithelial-to-mesenchymal transition in human prostate cancer cells. Cancer Res;70:6735-45

39. Carver BS, Tran J, Gopalan A, Chen Z, Shaikh S, Carracedo A*, et al.* Aberrant ERG expression cooperates with loss of PTEN to promote cancer progression in the prostate. Nat Genet 2009;41:619-24
40. Strittmatter BG, Jerde TJ, Hollenhorst PC. Ras/ERK and PI3K/AKT signaling differentially regulate oncogenic ERG mediated transcription in prostate cells. PLoS Genet;17:e1009708

41 J. Huang *et al*, The SIRT1 inhibitor EX-527 suppresses mTOR activation and alleviates acute lung injury in mice with endotoxiemia. Innate Immun,2017, 23;678-686

42 H.W Lim *et al*, SIRT1 deacetylates RORgammat and enhances Th17 cell generation. J Exp Med,2015 212, 607-17

Figure 1B

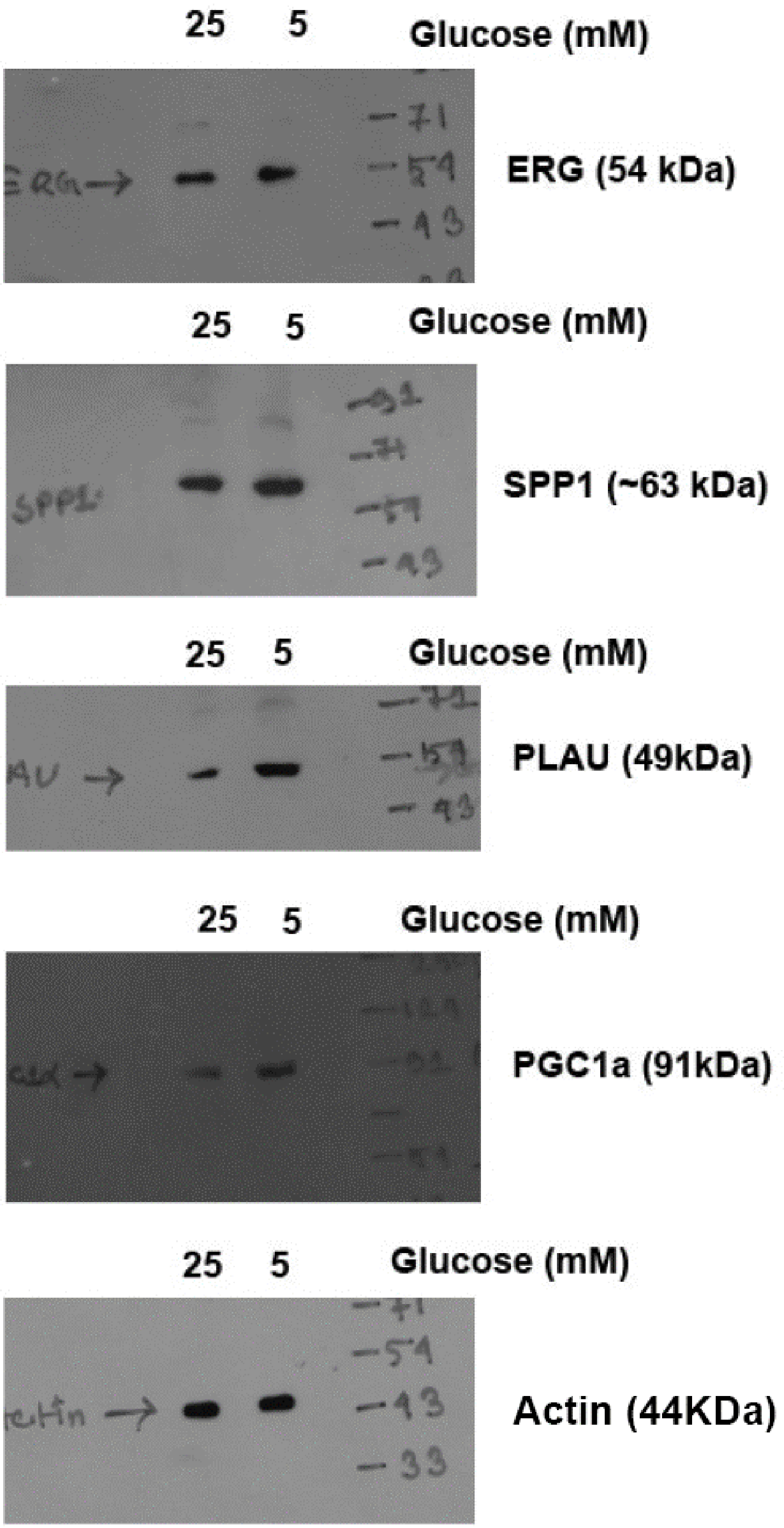
25
5
Glucose (mM)
ERG (54 kDa)
25
5
Glucose (mM)
SPP1 (~63 kDa)
25
5
Glucose (mM)
PLAU (49kDa)
25
5
Glucose (mM)
PGC1a (91kDa)
25
5
Glucose (mM)
Actin (44KDa)

Figure 1F

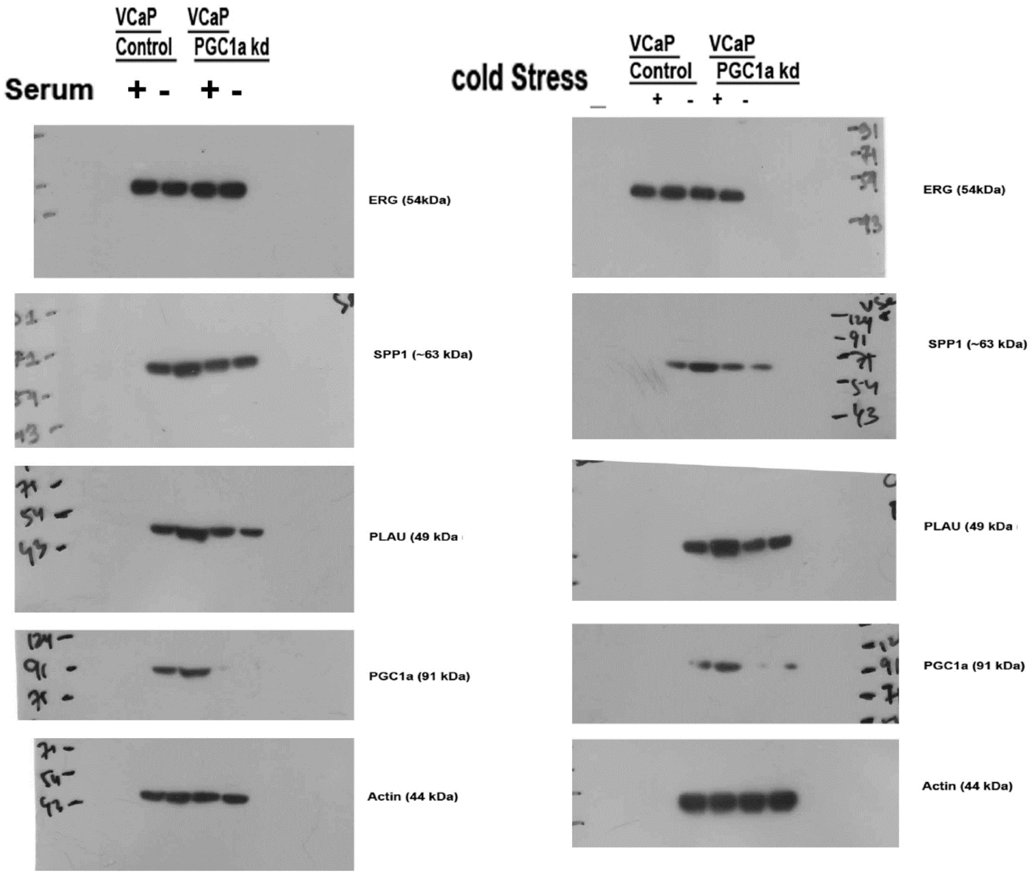
VCaP Control
VCaP PGC1a kd
Serum + - + -
cold Stress
VCaP Control
VCaP PGC1a kd
+ - + -
ERG (54kDa)
SPP1 (~63 kDa)
PLAU (49 kDa
PGC1a (91 kDa)
Actin (44 kDa)
ERG (54kDa)
SPP1 (~63 kDa)
PLAU (49 kDa
PGC1a (91 kDa)
Actin (44 kDa)

FIGURE: S1B

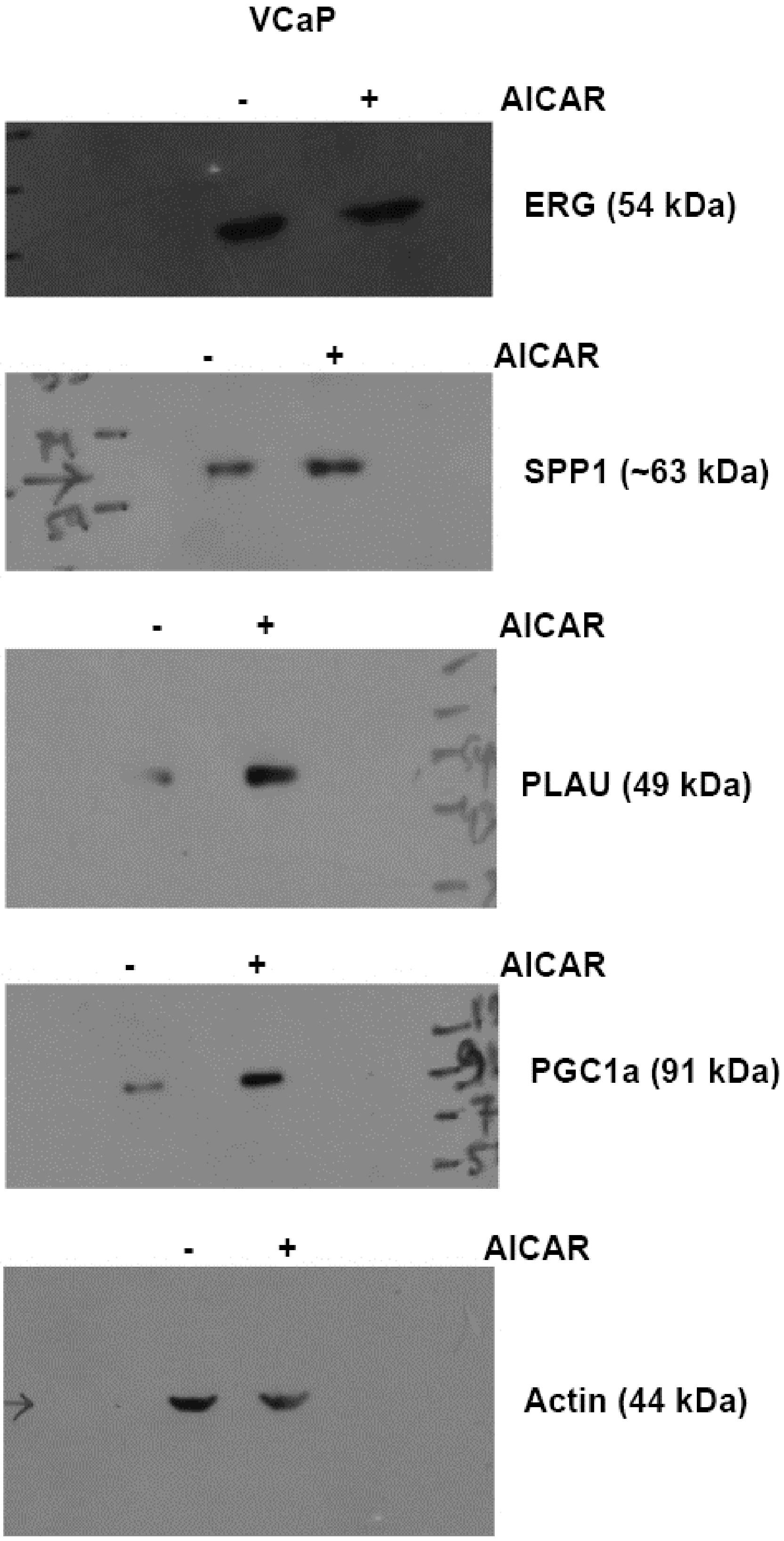
VCaP
- + AICAR
ERG (54 kDa)
- + AICAR
SPP1 (~63 kDa)
- + AICAR
PLAU (49 kDa)
- + AICAR
PGC1a (91 kDa)
- + AICAR
Actin (44 kDa)

FigureS1G

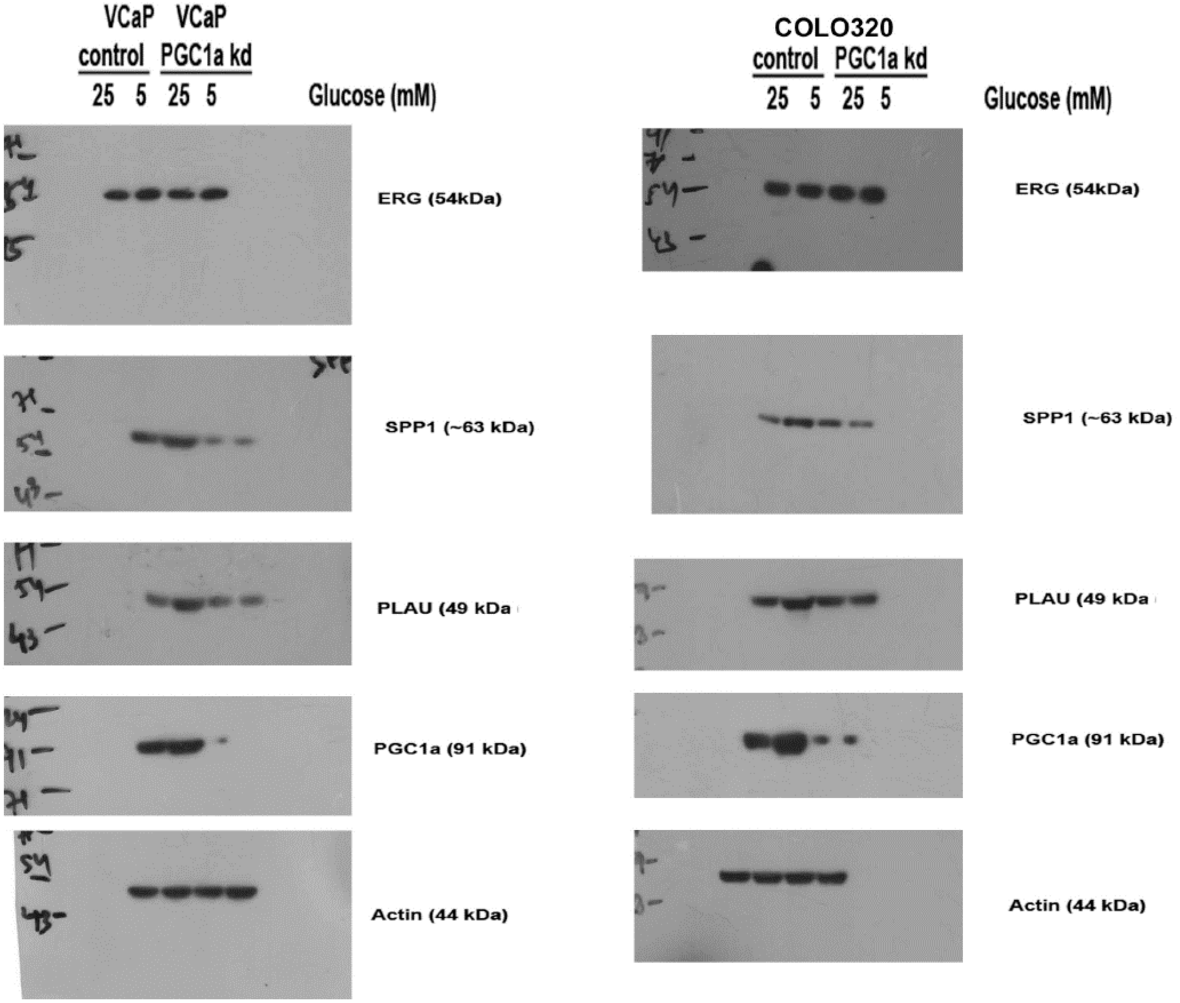
VCaP control
VCaP PGC1a kd
25 5 25 5
Glucose (mM)
ERG (54kDa)
SPP1 (~63 kDa)
PLAU (49 kDa
PGC1a (91 kDa)
Actin (44 kDa)
COLO320
control
PGC1a kd
25 5 25 5
Glucose (mM)
ERG (54kDa)
SPP1 (~63 kDa)
PLAU (49 kDa
PGC1a (91 kDa)
Actin (44 kDa)

Figure 2A

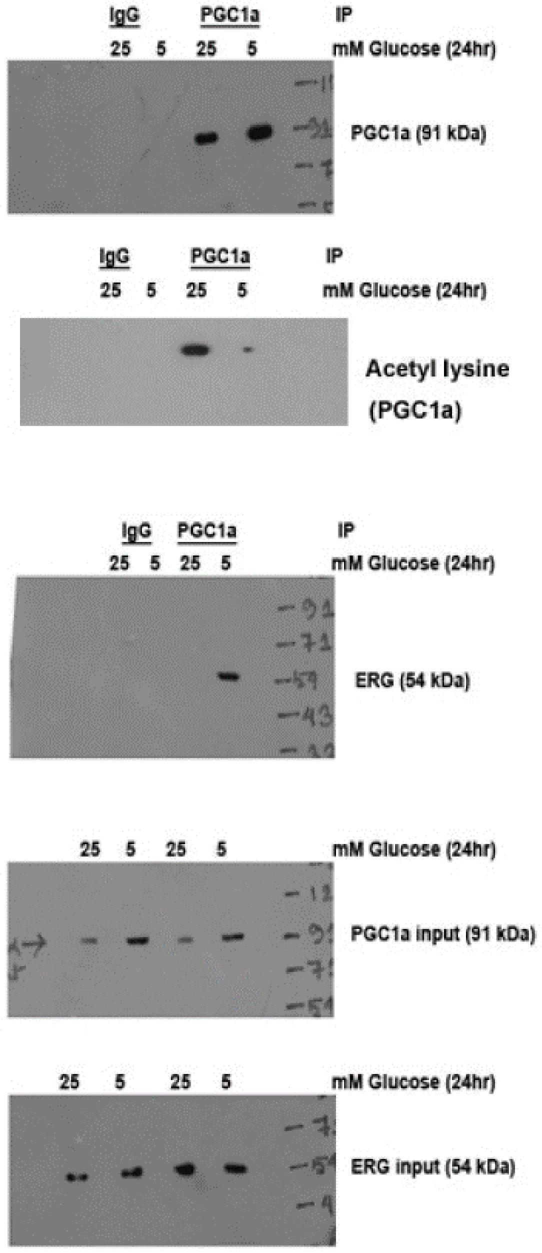

FIGURE 2B

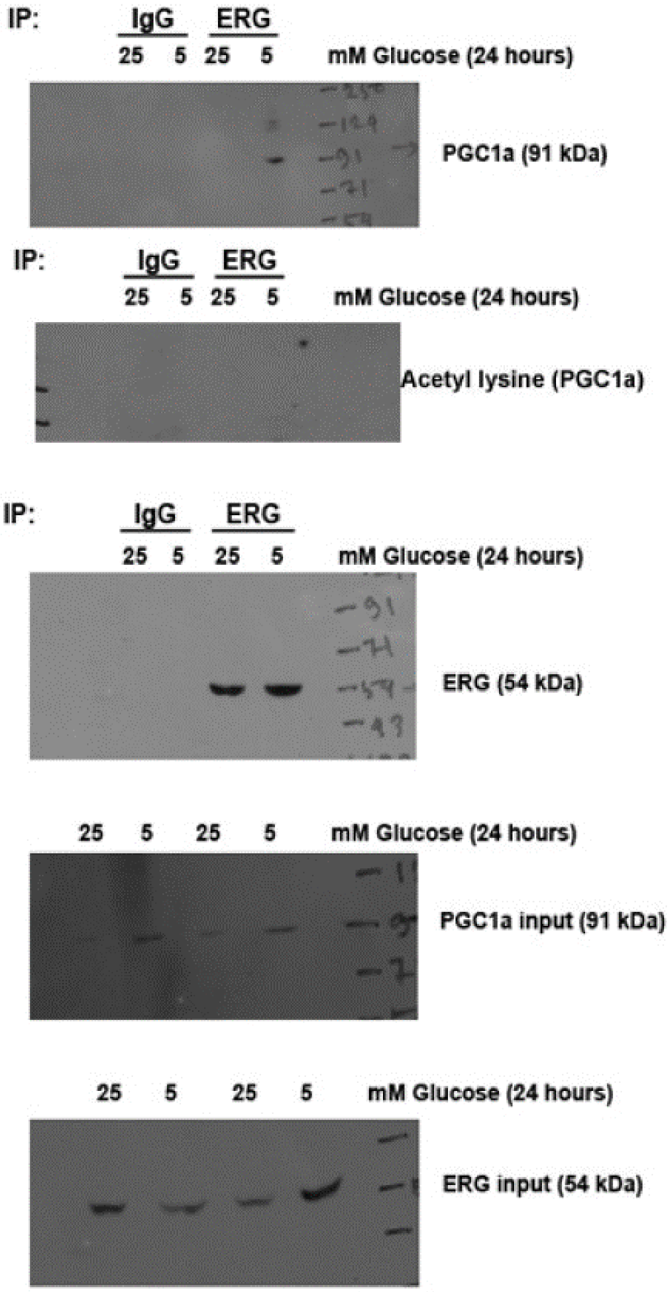

FIGURE: 2C

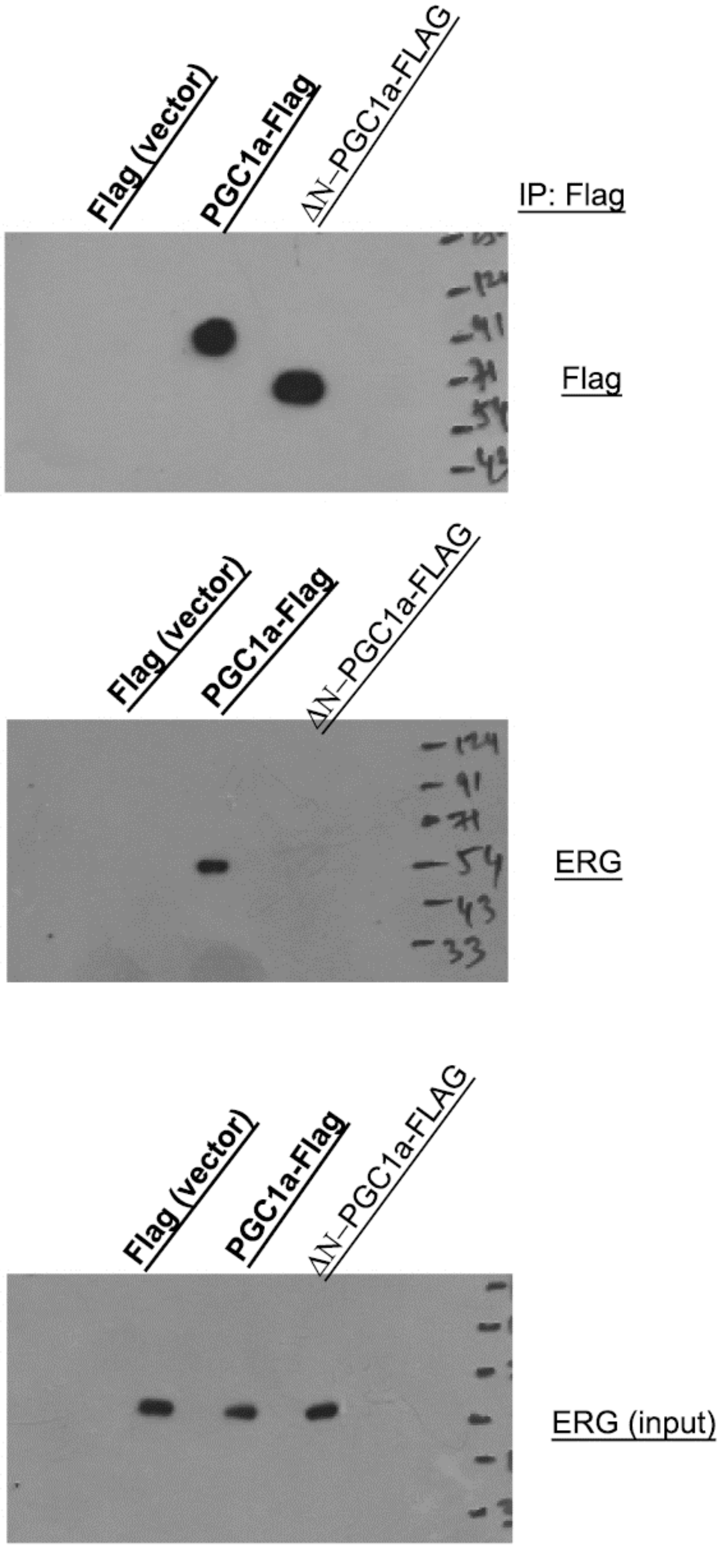
Flag (vector)
PGC1a-Flag
ΔN–PGC1a-FLAG
IP: Flag
Flag
Flag (vector)
PGC1a-Flag
ΔN–PGC1a-FLAG
ERG
Flag (vector)
PGC1a-Flag
ΔN–PGC1a-FLAG
ERG (input)

Figure S2A

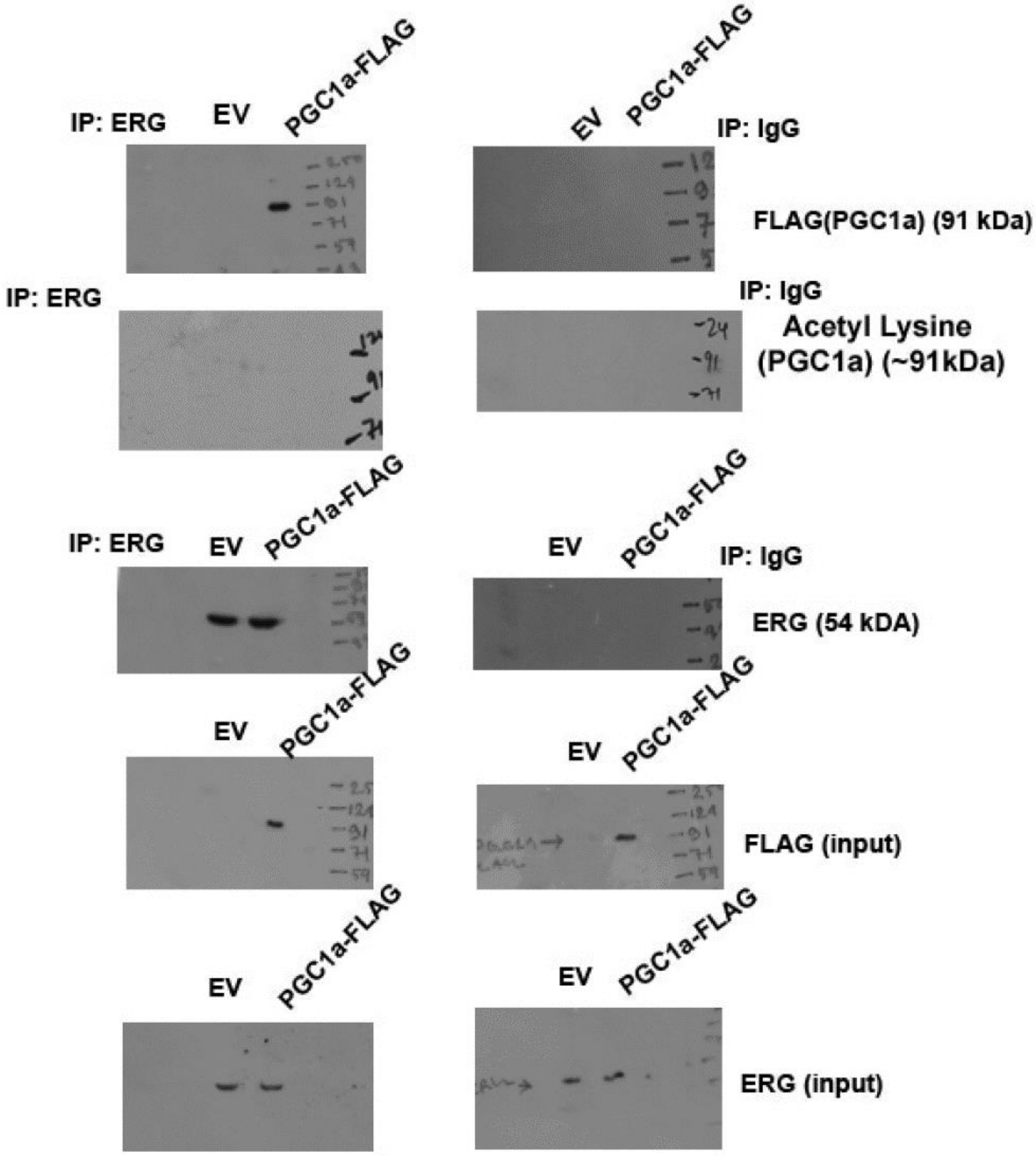

IP: ERG
EV
PGC1a-FLAG
EV
PGC1a-FLAG
IP: IgG
FLAG(PGC1a) (91 kDa)
IP: ERG
IP: IgG
Acetyl Lysine
(PGC1a) (~91kDa)
IP: ERG
EV
PGC1a-FLAG
EV
PGC1a-FLAG
IP: IgG
ERG (54 kDA)
EV
PGC1a-FLAG
EV
PGC1a-FLAG
FLAG (input)
EV
PGC1a-FLAG
EV
PGC1a-FLAG
ERG (input)

Figure S2B

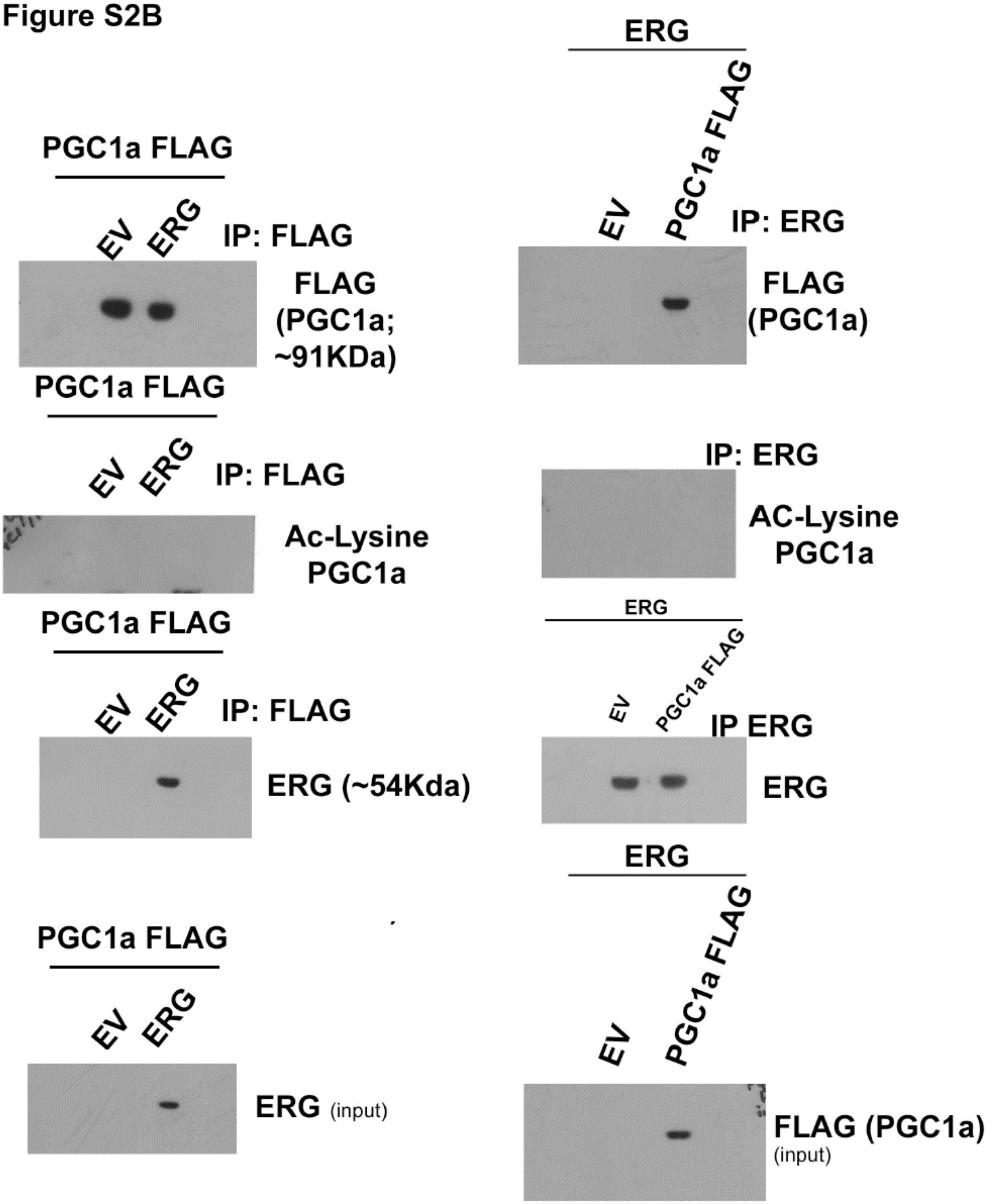
PGC1a FLAG
EV
ERG
IP: FLAG
FLAG (PGC1a; ~91KDa)
PGC1a FLAG
EV
ERG
IP: FLAG
Ac-Lysine PGC1a
PGC1a FLAG
EV
ERG
IP: FLAG
ERG (~54Kda)
PGC1a FLAG
EV
ERG
ERG (input)
ERG
EV
PGC1a FLAG
IP: ERG
FLAG (PGC1a)
IP: ERG
AC-Lysine PGC1a
ERG
EV
PGC1a FLAG
IP ERG
ERG
ERG
EV
PGC1a FLAG
FLAG (PGC1a) (input)

Figure S2C

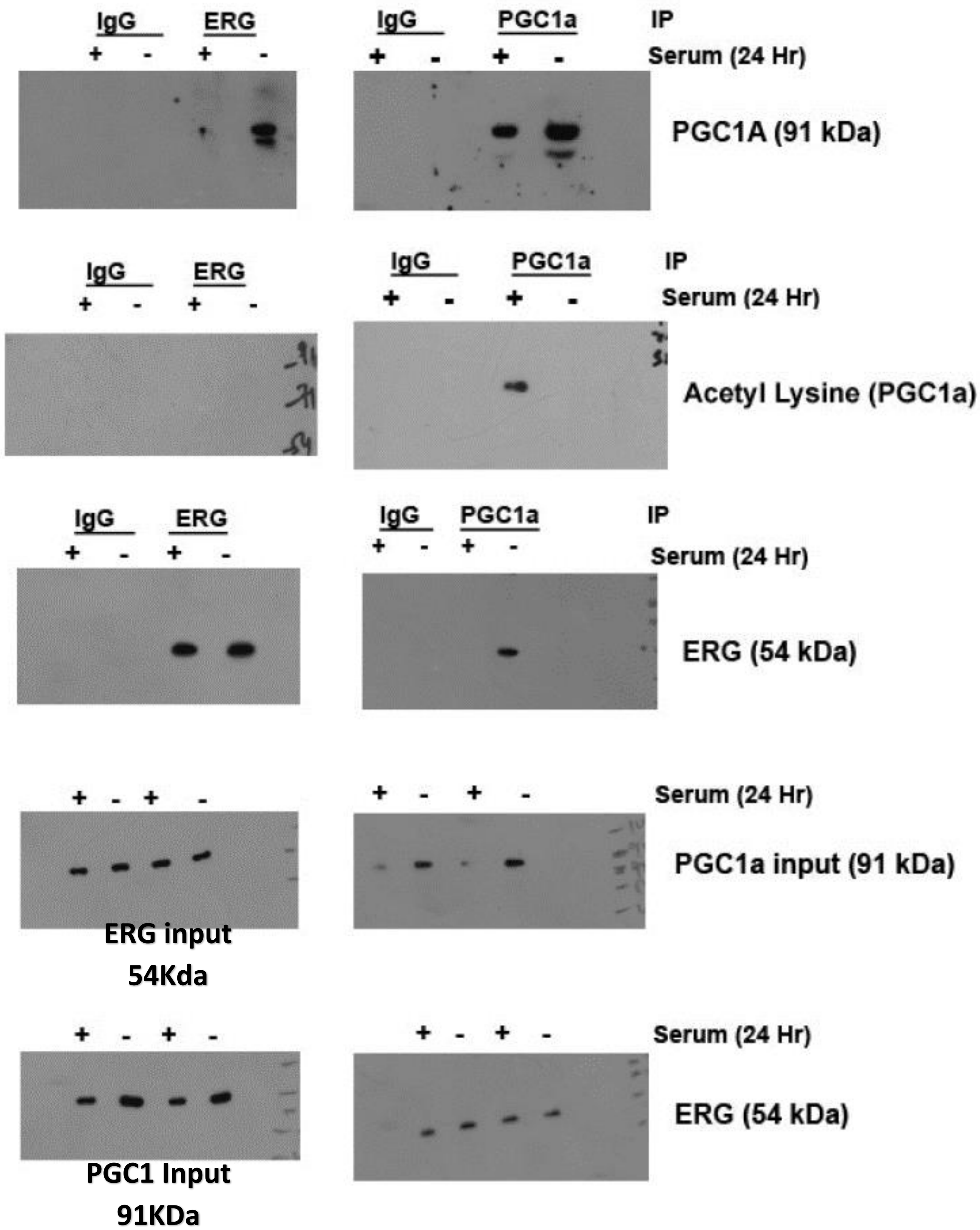

IgG
ERG
IgG
PGC1a
IP
+ - + -
+ - + -
Serum (24 Hr)
PGC1A (91 kDa)
IgG
ERG
IgG
PGC1a
IP
+ - + -
+ - + -
Serum (24 Hr)
Acetyl Lysine (PGC1a)
IgG
ERG
IgG
PGC1a
IP
+ - + -
+ - + -
Serum (24 Hr)
ERG (54 kDa)
+ - + -
+ - + -
Serum (24 Hr)
PGC1a input (91 kDa)
ERG input
54Kda
+ - + -
+ - + -
Serum (24 Hr)
ERG (54 kDa)
PGC1 Input
91KDa

Figure S2D

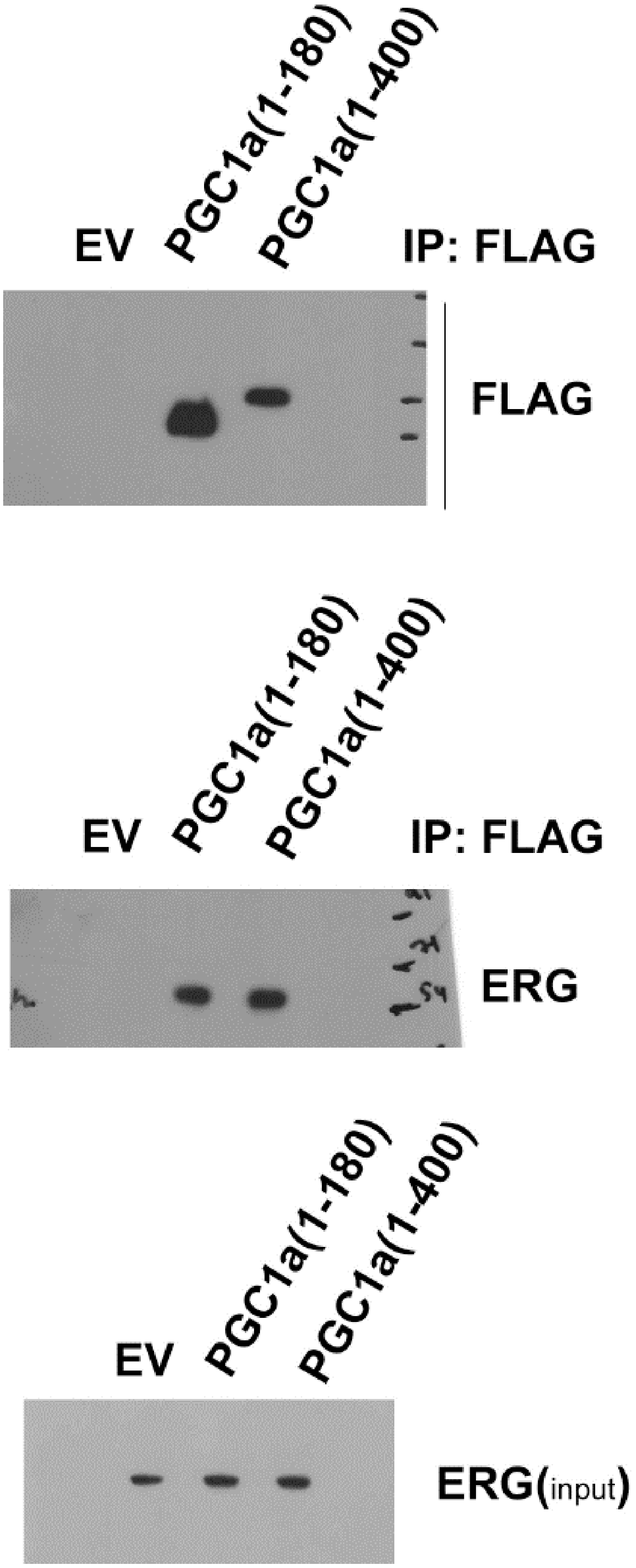
EV
PGC1a(1-180)
PGC1a(1-400)
IP: FLAG
FLAG
EV
PGC1a(1-180)
PGC1a(1-400)
IP: FLAG
ERG
EV
PGC1a(1-180)
PGC1a(1-400)
ERG(input)

FIGURE 3A

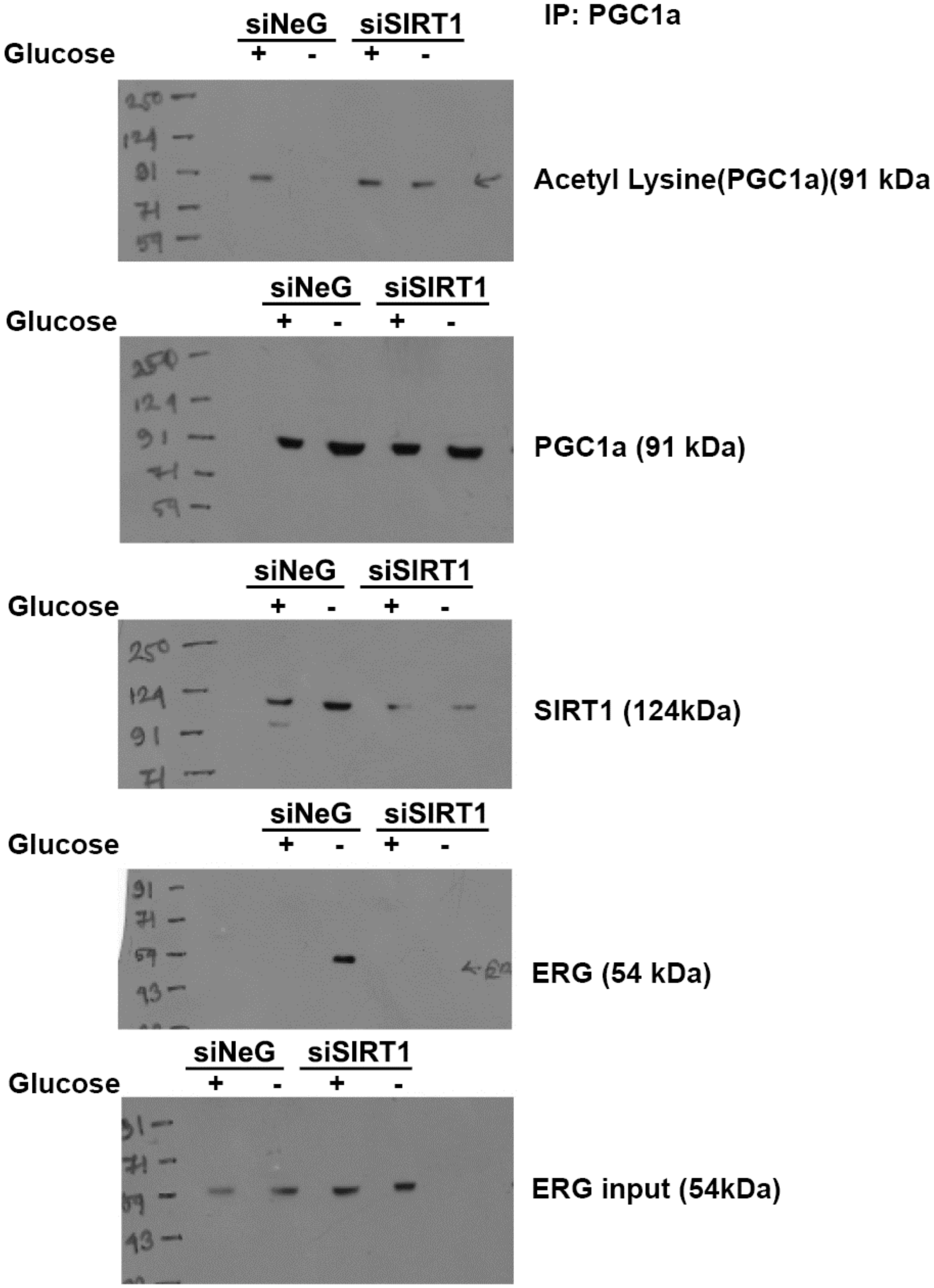

IP: PGC1a
siNeG
siSIRT1
Glucose
+ - + -
Acetyl Lysine(PGC1a)(91 kDa
PGC1a (91 kDa)
SIRT1 (124kDa)
ERG (54 kDa)
ERG input (54kDa)

FIGURE: 3B

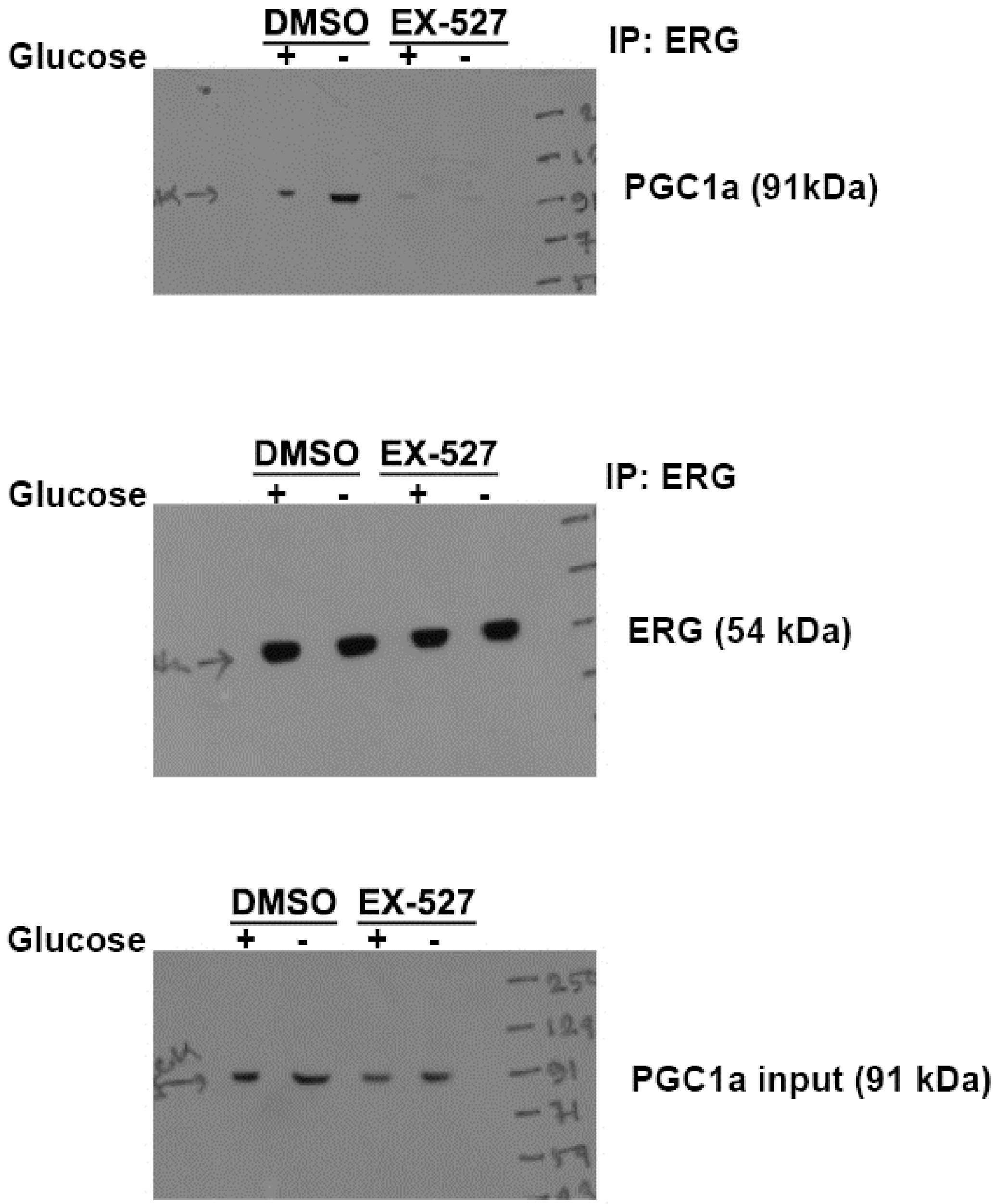
DMSO
EX-527
Glucose
+ - + -
IP: ERG
PGC1a (91kDa)
DMSO
EX-527
Glucose
+ - + -
IP: ERG
ERG (54 kDa)
DMSO
EX-527
Glucose
+ - + -
PGC1a input (91 kDa)
