# Supplementary material for: PGC1 alpha coactivates ERG fusion to drive antioxidant target genes under metabolic stress
Source: Commun Biol. 2022 May 4;5:416. doi: 10.1038/s42003-022-03385-x (PMC9068611; doi:10.1038/s42003-022-03385-x)
Supplement: Supplementary file 2 — Description of Additional Supplementary Files [file 42003_2022_3385_MOESM2_ESM.pdf]

Description of Additional Supplementary Files

File name: Supplementary Data 1
Description: Contains all normalized readings/data for main figures 1,2,3,4.

File name: Supplementary Data 2
Description: Contains all normalized readings/data for extended and supplementary figures S1,S2,S3,S4.
