# Supplementary material for: PGC1 alpha coactivates ERG fusion to drive antioxidant target genes under metabolic stress
Source: Commun Biol. 2022 May 4;5:416. doi: 10.1038/s42003-022-03385-x (PMC9068611; doi:10.1038/s42003-022-03385-x)
Supplement: Supplementary file 5 — Reporting Summary [file 42003_2022_3385_MOESM5_ESM.pdf]

nature portfolio

Corresponding author(s): DBPR, manuscript id:COMMSBIO-21-3085-T

Last updated by author(s): Mar 1, 2022

Reporting Summary

Nature Portfolio wishes to improve the reproducibility of the work that we publish. This form provides structure for consistency and transparency in reporting. For further information on Nature Portfolio policies, see our Editorial Policies and the Editorial Policy Checklist.

Statistics

For all statistical analyses, confirm that the following items are present in the figure legend, table legend, main text, or Methods section.

n/a	Confirmed	
☐	☒	The exact sample size (*n*) for each experimental group/condition, given as a discrete number and unit of measurement
☐	☒	A statement on whether measurements were taken from distinct samples or whether the same sample was measured repeatedly
☐	☒	The statistical test(s) used AND whether they are one- or two-sided<br>*Only common tests should be described solely by name; describe more complex techniques in the Methods section.*
☒	☐	A description of all covariates tested
☐	☒	A description of any assumptions or corrections, such as tests of normality and adjustment for multiple comparisons
☐	☒	A full description of the statistical parameters including central tendency (e.g. means) or other basic estimates (e.g. regression coefficient) AND variation (e.g. standard deviation) or associated estimates of uncertainty (e.g. confidence intervals)
☐	☒	For null hypothesis testing, the test statistic (e.g. *F*, *t*, *r*) with confidence intervals, effect sizes, degrees of freedom and *P* value noted<br>*Give P values as exact values whenever suitable.*
☒	☐	For Bayesian analysis, information on the choice of priors and Markov chain Monte Carlo settings
☒	☐	For hierarchical and complex designs, identification of the appropriate level for tests and full reporting of outcomes
☒	☐	Estimates of effect sizes (e.g. Cohen's *d*, Pearson's *r*), indicating how they were calculated

Our web collection on statistics for biologists contains articles on many of the points above.

Software and code

Policy information about availability of computer code

Data collection	data collected in microsoft excel office 2016, Microarray and sequencing data was obtained from NCBI GEO portal, PRAD patient data analysed over UCSC XENA browser
Data analysis	Data analysed over microsoft excel 2016, graphpad prism version 8, Integrated Genome Browser 9.0.1, Integrated genome viewer, Galaxy, Bowtie2, DESeq2, GSEA version 4, FlowJo v10.6,JASPER software

For manuscripts utilizing custom algorithms or software that are central to the research but not yet described in published literature, software must be made available to editors and reviewers. We strongly encourage code deposition in a community repository (e.g. GitHub). See the Nature Portfolio guidelines for submitting code & software for further information.

Data

Policy information about availability of data

All manuscripts must include a data availability statement. This statement should provide the following information, where applicable:

- Accession codes, unique identifiers, or web links for publicly available datasets
- A description of any restrictions on data availability
- For clinical datasets or third party data, please ensure that the statement adheres to our policy

The following data sets are used for GSEA analysis GSE16671, GSE110656 ,GSE14595; GSE164859. For ChIP sequencing data analysis GSE110655, GSE14092, GSE28950 were used. All datasets used for the study are listed in supplementary table4. Other online tools like UCSC XENA browser used TCGA PRAD and GTex data, SURV express used TCGA prostate cancer and GSE40272 datasets.

Field-specific reporting

Please select the one below that is the best fit for your research. If you are not sure, read the appropriate sections before making your selection.

☒ Life sciences ☐ Behavioural & social sciences ☐ Ecological, evolutionary & environmental sciences

For a reference copy of the document with all sections, see nature.com/documents/nr-reporting-summary-flat.pdf

Life sciences study design

All studies must disclose on these points even when the disclosure is negative.

Sample size	No sample size was decided initially. However, similar experimental conditions across biological replicates were used for statistical significance. In vitro experiments were performed with atleast 2 technical replicates everytime the experiment is done and has been repeated atleast to have biological triplicates as mentioned in respective figure legends
Data exclusions	no data was excluded from analysis.
Replication	majority of experiments were repeated multiple times with independent biological samples using similar experimental conditions and is mentioned in legends, methods and text.
Randomization	for in vivo experiments mice were randomized before injecting cells for xenografts.<br>for invitro treatments cell plates/ culture dishes were randomly assigned control or treatment groups before starting experiments
Blinding	Investigators couldn't be blinded in well defined treatment and control group experiment during data collection and analysis. However, investigators were blinded to sample identity during IHC of tumor sections.

Reporting for specific materials, systems and methods

We require information from authors about some types of materials, experimental systems and methods used in many studies. Here, indicate whether each material, system or method listed is relevant to your study. If you are not sure if a list item applies to your research, read the appropriate section before selecting a response.

Materials & experimental systems

n/a	Involved in the study	
☐	☒	Antibodies
☐	☒	Eukaryotic cell lines
☒	☐	Palaeontology and archaeology
☐	☒	Animals and other organisms
☒	☐	Human research participants
☒	☐	Clinical data
☒	☐	Dual use research of concern

Methods

n/a	Involved in the study	
☒	☐	ChIP-seq
☒	☐	Flow cytometry
☒	☐	MRI-based neuroimaging

Antibodies

Antibodies used	PGC1a (Thermofisher, PA572948)<br>Spp1 (Abcam, Ab181440)<br>PLAU (Abcam, Ab133563)<br>OctA-probe(G-8)/ FLAG (Santa cruz biotechnology, sc166384)<br>ERG (abcam, Ab133264)<br>Acetyl Lysine (Cell Signaling Technology, 9441)<br>Sirt1 (abcam, ab110304)<br>Anti rabbit (BioRad, 170-6515)<br>Anti mouse (Cell Signaling Technology, 7076S)<br>Beta-actin (ThermoFisher, MA5-15739)<br>Normal Rabbit IgG (abcam, Ab171870)<br>Cleaved caspase-3 cleavage (CC3) (Cell Signaling Technology, 9661)
Validation	1. PA572948: Immunogen: Synthetic peptide made to an internal portion of the human PGC-1 alpha protein. Applications: WB, IP, RIP.<br>2. Ab181440: Immunogen: Synthetic peptide corresponding to Human Osteopontin (N terminal). Applications: WB, IHC-P<br>3. Ab133563: Immunogen: Synthetic peptide within Human Urokinase. Applications: WB, IHC-P<br>4. sc166384: raised against OctA (FLAG)-tagged proteins. Applications: WB, IP, IF and ELISA<br>5. Immunogen: Synthetic peptide within Human ERG. Applications: Flow Cyt, ICC/IF, WB, IP, IHC-p

6. Acetylated-Lysine Antibody detects proteins post-translationally modified by acetylation on the epsilon-amine groups of lysine residues. Applications: WB, IP, IHC, ChIP, IF,
7. ab110304: Immunogen: Recombinant Human SIRT1. Applications: WB, Flow Cyt, ICC/IF, IHC-P
8. biorad 170-6515: Secondary HRP-conjugated anti-rabbit.
9. CST 7076S: Anti-mouse IgG, HRP-linked Antibody. Applications: WB, IP, IHC, ChIP, IF, ELISA
10. Immunogen: Beta-actin N-terminal peptide. Applications: WB, IHC-P, IP, ELISA
11. Ab171870: Rabbit IgG, polyclonal, isotype control. Applications: WB, ChIP, Flow Cyt
12. CST#9661: Cleaved Caspase-3 (Asp175) Antibody detects endogenous levels of the large fragment (17/19 kDa) of activated caspase-3 resulting from cleavage adjacent to Asp175. This antibody does not recognize full length caspase-3 or other cleaved caspases. Application: WB, IP, IHC, Flow Cytometry

Eukaryotic cell lines

Policy information about cell lines

Cell line source(s)	VCaP (ATCC:CRL-2876™) purchased from ATCC. PC3 and COLO320 cells was purchased from National center for cell sciences (Pune, India) and cultured as recommended. shRNA stable cells were generated inhouse from parental VCaP cells or parental COLO320 cells purchased from above sources.
Authentication	Cell lines were authenticated by short tandem repeat (STR) profiling at NCCS PUNE, India.
Mycoplasma contamination	All cell lines were determined to be mycoplasma free using the MycoAlert Plus system and kit (Lonza)
Commonly misidentified lines (See ICLAC register)	no misidentified cell lines were used in this study

Animals and other organisms

Policy information about studies involving animals; ARRIVE guidelines recommended for reporting animal research

Laboratory animals	SCID mice NOD.Cg-Prkdcscid (The Jackson Laboratory) strain, all Male, were obtained from breeding colony maintained by institutional animal facility and were handled in pathogen-free conditions. Xenograft formation was conducted using mice of 4-6 weeks of age
Wild animals	no wild animals used in study
Field-collected samples	no samples collected from field study
Ethics oversight	All mice experiments were approved by institutional animal ethical committee and abide by all regulatory standard of Rajiv Gandhi Centre for Biotechnology (RGCB), Trivandrum

Note that full information on the approval of the study protocol must also be provided in the manuscript.
